# Putative pathogen-selected polymorphisms in the *PKLR* gene are associated with mycobacterial susceptibility in Brazilian and African populations

**DOI:** 10.1371/journal.pntd.0009434

**Published:** 2021-08-27

**Authors:** Ohanna Cavalcanti de Lima Bezerra, Lucia Elena Alvarado-Arnez, Nédio Mabunda, Graça Salomé, Amina de Sousa, Fernanda de Souza Gomes Kehdy, Carolinne Sales-Marques, Fernanda Saloum de Neves Manta, Rafaela Mota Andrade, Laís Pereira Ferreira, Thyago Leal-Calvo, Cynthia Chester Cardoso, Kelly Nunes, Mateus H. Gouveia, Sam M. Mbulaiteve, Edward D. Yeboah, Ann Hsing, Ana Carla Pereira Latini, André Luiz Leturiondo, Fabíola da Costa Rodrigues, Ariani Batista Noronha, Cynthia de Oliveira Ferreira, Carolina Talhari, Jamile Leão Rêgo, Léa Cristina de Carvalho Castellucci, Eduardo Tarazona-Santos, Elizeu Fagundes de Carvalho, Diogo Meyer, Roberta Olmo Pinheiro, Ilesh V. Jani, Antonio Guilherme Pacheco, Milton Ozório Moraes

**Affiliations:** 1 Laboratory of Leprosy, Oswaldo Cruz Institute, FIOCRUZ, Rio de Janeiro, Brazil; 2 National Research Coordination, Franz Tamayo University (UNIFRAZ), Cochabamba, Bolivia; 3 Laboratory of Molecular Virology, Instituto Nacional de Saúde, Maputo, Mozambique; 4 Medical Faculty, Eduardo Mondlane University, Maputo, Mozambique; 5 Laboratory of Cellular Biology and Genetics, Federal University of Alagoas, Arapiraca, Brazil; 6 Laboratory of Molecular Virology, Department of Genetics, Federal University of Rio de Janeiro, Rio de Janeiro, Brazil; 7 Laboratory of Evolutionary Genetics and Biology, Biosciences Institute, University of São Paulo, São Paulo, Brazil; 8 Center for Research on Genomics and Global Health, National Human Genome Research Institute, National Institutes of Health, Bethesda, Maryland, United States of America; 9 University of Ghana Medical School, Accra, Ghana; 10 Stanford Cancer Institute, Stanford University, Stanford, California, United States of America; 11 Sector of Pharmacology, Lauro de Souza Lima Institute (ILSL), Bauru, Brazil; 12 Laboratory of Molecular Biology, Alfredo da Matta Foundation, Manaus, Brazil; 13 Immunology Service, Professor Edgard Santos University Hospital, Federal University of Bahia, Salvador, Brazil; 14 Departament of Biology, Institute of Biological Sciences, Federal University of Minas Gerais (UFMG), Belo Horizonte, Brazil; 15 DNA Diagnostic Laboratory (LDD), Rio de Janeiro State University (UERJ), Rio de Janeiro, Brazil; 16 Science Computational Program (PROCC), FIOCRUZ, Rio de Janeiro, Brazil; University of Pittsburgh, UNITED STATES

## Abstract

Pyruvate kinase (PK), encoded by the *PKLR* gene, is a key player in glycolysis controlling the integrity of erythrocytes. Due to *Plasmodium* selection, mutations for PK deficiency, which leads to hemolytic anemia, are associated with resistance to malaria in sub-Saharan Africa and with susceptibility to intracellular pathogens in experimental models. In this case-control study, we enrolled 4,555 individuals and investigated whether *PKLR* single nucleotide polymorphisms (SNPs) putatively selected for malaria resistance are associated with susceptibility to leprosy across Brazil (Manaus–North; Salvador–Northeast; Rondonópolis–Midwest and Rio de Janeiro–Southeast) and with tuberculosis in Mozambique. Haplotype T/G/G (rs1052176/rs4971072/rs11264359) was associated with leprosy susceptibility in Rio de Janeiro (OR = 2.46, *p* = 0.00001) and Salvador (OR = 1.57, *p* = 0.04), and with tuberculosis in Mozambique (OR = 1.52, *p* = 0.07). This haplotype downregulates *PKLR* expression in nerve and skin, accordingly to GTEx, and might subtly modulate ferritin and haptoglobin levels in serum. Furthermore, we observed genetic signatures of positive selection in the *HCN3* gene (xpEHH>2 –recent selection) in Europe but not in Africa, involving 6 SNPs which are *PKLR/HCN3* eQTLs. However, this evidence was not corroborated by the other tests (F_ST_, Tajima’s D and iHS). Altogether, we provide evidence that a common *PKLR* locus in Africans contribute to mycobacterial susceptibility in African descent populations and also highlight, for first, *PKLR* as a susceptibility gene for leprosy and TB.

## Author summary

Investigation of human genes under pathogen-driven selection as *Plasmodium* sp. has pinpointed genetic variants that participate in the adaptation to the environment and/or are related to severities of human diseases. The current study examined an example of an evolutionary trade-off in which genetic variants in the *PKLR* gene putatively selected for malaria resistance influence the susceptibility to mycobacterial diseases (leprosy and tuberculosis) in Brazilian population and Mozambique. A complete characterization of the biological effect of those risk variants may clarify the role of the *PKLR* gene in leprosy and tuberculosis. Deciphering the genetic basis of mycobacterial diseases has implications for the identification of true high-risk individuals in order to optimize screening strategies. Furthermore, the trade-off mechanism discussed in this work might occur in other central genes of immune response and biochemical pathways, controlling the susceptibility to other infectious diseases.

## Introduction

Infectious diseases are one of the most important selective forces driving the genetic variation in humans [1,2]. Throughout human co-evolution with microorganisms in distinct geographical areas, different alleles may have been selected. Thus, as populations evolve in response to distinct local selective pressures, the set of selected genetic variants may differ between them [3,4]. Depending on the mode and intensity, it is possible to classify natural selection into different regimes: positive selection (increases the frequency of advantageous variants), purifying (eliminating deleterious variants) and balancing (a set of regimes that have in common the maintenance of genetic variants at intermediate frequency in the population). One of the classic examples of balancing selection is the augmented frequency of the HbS allele for sickle cell disease in Sub-Saharan Africans, where malaria is endemic. Although HbS homozygous individuals have severe anemia and a number of clinical complications related to sickling of red blood cells (RBCs), those who are heterozygous have a survival advantage as it confers resistance to *Plasmodium* sp. [5]. This demonstrates the maintenance of a specific phenotype due to pathogen-driven selection [6]. As malaria kills millions of individuals annually, mostly affecting children below 5 years, those with protective mutations against *Plasmodium* are more likely to be selected [7–9], thus, the number of reported malaria-protective polymorphisms has been increasing. Most of these polymorphisms affect structural and enzyme-related erythrocyte genes, such as *G6PD* (glucose-6-phosphate dehydrogenase deficiency), hemoglobinα and β genes (thalassemia) and *ATP2B4* (Burkitt lymphoma) [10–13]. Likewise, other clinically silent mutations may have been under positive or balancing selection due to their ability to provide survival advantage against *Plasmodium* infection.

*PKLR* encodes for pyruvate kinase (PK), an enzyme that converts phosphoenolpyruvate into pyruvate and ATP in the last step of glycolysis. Defects in this enzyme are the most common cause of hereditary non-spherocytic hemolytic anemia, as it reduces ATP levels and decreases the erythrocyte lifespan [14,15]. Mutations for PK deficiency have been reported as conferring protection against malaria in murine and *in vitro* models [16–19]. In human population, genetic screens have also suggested that mutations for PK deficiency have been selected due to their protective effect against the *Plasmodium* infection in malaria-endemic areas [20,21] and there have been claims that the *PKLR* locus is under selective pressure in African populations [22–24]. However, further studies involving larger and more diverse samples and denser sets of markers are required to confirm this hypothesis. Indeed, *PKLR* SNPs are at considerably higher frequencies in African than in Portuguese populations compared with neutral markers [22,25]. In addition, a strong linkage disequilibrium (LD) between *PKLR* and adjacent loci within individuals with no malaria infection or non-complicated malaria suggested a conserved genomic region probably selected due to some level of malaria protection [22]. However, the biological effect of these selected variants may also be associated to other phenotypes with reduced fitness. Mutations for PK deficiency have been associated with susceptibility to the intracellular pathogen *Salmonella typhimurium* due to iron overload inside macrophages in murine models [26]. The product of RBC lysis–heme–is processed and iron is recycled within macrophages. Given that iron is crucial for the survival of intracellular pathogens, a supply of this element could favor their multiplication [27]. Therefore, *PKLR* polymorphisms may display functional variation in erythrocyte lysis or iron homeostasis and hence increase the susceptibility to other intracellular pathogens [26], such as *Mycobacterium leprae* and *M*. *tuberculosis*. Leprosy and tuberculosis (TB) still remain public health concerns worldwide, particularly in Brazil. For this reason, deciphering the genetic susceptibility of complex diseases could clarify why they are able to persist in certain populations [28,29].

Brazil is an admixed country derived from three main well-defined ancestral sources: Europeans, Africans and Native-Americans [30,31]. Considering that African populations have patterns of genetic variation indicative of selection on the *PKLR* gene, presumably historically-driven by heavy exposure to *Plasmodium* [21–23,25], Brazilian populations may also carry this genomic signature, since African ancestry is a significant part of their genetic makeup. Given the possibility that genetic variations in the *PKLR* gene may influence the susceptibility to other intracellular pathogens, we investigated whether *PKLR* SNPs, possibly selected for *Plasmodium* resistance in Africa, are associated with susceptibility to mycobacterial diseases. Here, we present a retrospective association study with leprosy in representative populations from the Southeast, Midwest, North and Northeast of Brazil, and with TB in an African population. We consistently confirmed a modest susceptibility association of the haplotype T/G/G (rs1052176/rs4971072/rs11264359) with leprosy in Brazil, particularly in sites with higher African content, Rio de Janeiro and Salvador, and with TB in Mozambique. The haplotype T/G/G was also related with a decrease in *PKLR* gene expression, accordingly to GTEx, and might alter ferritin and haptoglobin measurements, which could have implications in the susceptibility to mycobacterial diseases. Furthermore, we used public genomic data from the 1000 Genomes Project to test for natural selection in the *PKLR* genomic region. We observed a signal of selective sweep (xpEHH test) in regions containing eQTL for *PKLR* and *HCN3* genes in Europeans, contrary to the suggestive balancing selection observed by the intermediate allele frequencies in Africa. This indicates that the selective mechanisms operating in this genomic region are complex and may had varied over time, requiring further investigations.

## Materials and methods

### Ethics statement

All collected samples and procedures described in this study were approved by local ethics boards and the Brazilian National Board for Ethics in Research. A written informed consent was obtained from all voluntary participants (Rio de Janeiro–IRB protocol–Fiocruz 151/01; Rondonópolis–ILSL 172/09; Salvador–CEP50/2010 and CONEP 11019; Manaus– 555.620–13/03/2013; and Mozambique–N° 399/CNBS/11).

### Genomic data collection and SNPs selection

The population used to define tag SNPs in the *PKLR* gene region included 504 Africans (ENS, GWD, LWK, MSL and YRI), 503 Europeans (CEU, FIN, GBR, IBS and TSI) and 347 Native Americans from the publicly available genomic data of the 1000 Genomes Project phase III [32]. The step-by-step diagram of the SNPs selection is stated in S1A Fig and an expansion of the methods’ details is described in S1 Appendix. First, we calculated the allele frequency of the SNPs from a region of 10,000 bp *upstream* and *downstream* of the gene *loci* (chr1:155.259.084–155.271.225 –GRCh37/hg19), with a total length of ≈ 32 Kb. Next, Principal Component Analysis (PCA) was performed using EIGENSOFT 4.2 [33] and it was the first filter applied to select the SNPs. PCA is used for population stratification in genetic studies. Here, we have adapted the use of PCA for guiding the SNPs selection considering that the divergence in allelic/genotype frequency among populations would be driven, at least in part, by events of natural selection [34]. We used only the variants within and around of the *PKLR* genomic region (921 variants were used as input) and hypothesized that the different clusters could be guided by “top SNPs” with a functional role at the gene (S1B Fig). In this case, SNPs with the highest “weights” had the higher capacity of clustering into groups in the principal component 1 (PC1), which captures the overall variability of the variants in the intragenic region. In S1B Fig, PC1 does not separate European from African and Native-American populations, suggesting that ancestry is not the main factor influencing genetic variability at the gene. Since we are selecting variants within a unique gene, we hypothesized that the functional variants would guide this clustering. Then, we selected the thirty variants with the highest values of “SNP weight” for the PC1, which we called “top SNPs”, and kept only the SNPs with minor allele frequency (MAF) above 10%. The top SNPs based on the PC1 have high-differentiated allele frequencies among populations and might have a degree of LD. Then, to define tag SNPs, linkage disequilibrium (LD) analysis and haplotype inferences in Europeans and Africans were analyzed by HAPLOVIEW [35]. In addition, the three cohorts from the EPIGEN-Brazil Initiative consortium (90 healthy individuals from Salvador, 88 from Bambuí and 87 from Pelotas) (https://epigen.grude.ufmg.br) were assessed to compare the allele and haplotype frequencies in the Brazilian population [36–38]. Finally, SNPs were annotated by ANNOVAR [39] with refGene hg19 (11 Dec 2015). Thus, (1) starting with the PCA filtering, we compared each of the “30 top SNPs” chosen by the values of “SNP weight” in the PC1 with the next analyses and kept the SNPs meeting at least three of the following conditions: (2) present MAF>10% in Africans or Europeans, (3) were not in high LD (r^2^>0.8 was considered to define SNPs in high LD) among them and displayed divergent haplotype inferences between Europeans and Africans, (4) were localized in coding/regulatory regions by ANNOVAR, and (5) were reported as associated in malaria studies were selected [23,25]. The literature was used to include variants previously related with PK deficiency and malaria selection. Thus, three SNPs were selected for the association studies: rs1052176 (exonic), rs4971072 (intergenic) and rs11264359 (intronic) (S1 Table). The three variants tag blocks covering the genomic region, as observed in the LD plot of Africans and Europeans in S2 Fig.

### Case-control studies

Details of the 4,555 individuals in each of the five studied populations (four regions of Brazil and Mozambique, East Africa) are given in S2 Table, in published reports [40–43] and are available at Zenodo repository (https://doi.org/10.5281/zenodo.3876692). We performed two case-control studies: an association study with leprosy in Rio de Janeiro (Southeast Brazil) followed by a replication in three Brazilian cohorts of Rondonópolis (State of Mato Grosso, Midwest Brazil), Salvador (State of Bahia, Northeast Brazil) and Manaus (State of Amazonas, North Brazil), and a second independent association study with tuberculosis in Mozambique. For the initial study in Rio de Janeiro, DNA from 777 leprosy cases from the Souza Araújo Ambulatory (ASA) at FIOCRUZ (Rio de Janeiro, Brazil) and 597 unrelated healthy bone marrow donors from National Institute of Cancer (INCA) (Rio de Janeiro, Brazil) were used. Leprosy diagnosis was confirmed by clinical, histological, serological and molecular tests. The replication study in Rondonópolis enrolled 411 leprosy cases treated in government healthcare clinics and 358 controls matched to the case group according to epidemiological characteristics (ethnicity, gender, age and geographical region). Samples from Salvador included 238 leprosy cases diagnosed at the Hospital Edgard Santos and Dom Rodrigo de Menezes (Salvador, Brazil), reference centers for treatment of the disease, and 312 local blood donors recruited as controls. Lastly, 424 leprosy patients and 967 controls from Manaus were recruited at the Alfredo da Matta Foundation (Manaus, Brazil). Controls were individuals who lived in the same endemic area as the cases whose exams had no suspected leprosy lesions and declared no previous contact with leprosy or TB patients. Diagnosis was obtained by clinical, slit skin smears and histopathological findings. All leprosy patients were classified according to the Ridley and Jopling [44] and World Health Organization classifications (paucibacillary or multibacillary) [28,29]. Furthermore, 471 participants were included in the Mozambique case-control study: 104 pulmonary tuberculosis cases (PTB) recruited in Mavalane, Polana Caniço and Machava Hospitals and 367 controls without previous history of TB infection. None of the TB individuals were positive for the human immunodeficiency virus.

### SNPs genotyping assay

DNA samples were extracted from peripheral blood according to the salting-out method [45]. Total amount of nucleic acids (10–40 ng) and purity were measured by NanoDrop ND-1000 (Thermo Fisher). All samples were genotyped by fluorescence-based allelic discrimination using TaqMan Genotyping Assay in the StepOnePlus 2.1 Real-Time PCR (Thermo Fisher). All reactions were run in a final volume of 5 μL (2.5 μL of TaqMan Genotyping Master Mix and 0.125 μL of TaqMan primers and probes).

### Ancestry analysis on population structure

All Brazilian cohorts were genotyped for a panel of 46 autosomal ancestry informative markers (46 AIM-Indels) of type INDEL by a single multiplex PCR followed by capillary electrophoresis, according to the protocol described previously [46]. The reaction volume was 5 μL using 0.5 to 5 ng/μL of template DNA. Dye-labeled amplified fragments were separated and detected using an ABI 3500 Genetic Analyzer (Thermo Fisher). PCR thermocycling conditions were an initial step of 15 min at 95°C, followed by 27 cycles at 94°C for 30 s, 60°C for 1.5 min, 72°C for 45 s and a final extension at 72°C for 60 min. Automated allele calls were obtained by GeneMapper v.4.1 (Thermo Fisher). Genetic ancestry for each of the different Brazilian cohorts studied was estimated by STRUCTURE v2.3.3 [47]. We performed a supervised analysis using prior information on the geographic origin of the reference samples. Considering the historically formation of Brazilians, we assumed an essentially tri-hybrid ancestral contribution from Native-Americans, Europeans and Africans (i.e., K = 3). Data of these three populations from the HGDP-CEPH diversity panel (sub-set H952) were used as reference (ancestral populations). We selected the option “*Use population information to test for migrants*” with the “*Admixture model*” for the runs; allele frequencies were correlated and updated using only individuals with POPFLAG = 1. Data of the ancestry estimates (S2 Table and S3 Fig) were used to control for population structure in the association studies.

### Functional study individuals and serum measurements

The population included in the genotype–phenotype analysis was a combination of newly collected samples from 150 healthy volunteers and 141 leprosy patients from FIOCRUZ diagnosed as reported previously. Healthy participants included 106 women and 44 men (mean age 32.1±8.7 years). In the patient group, 48 were women and 93 men (mean age 45.2±15.7 years). Leprosy patients were classified according to Ridley and Jopling and cases with reaction stages were excluded from the analysis. Individuals declared no history of infectious diseases, alcoholism or hepatic/metabolic pathologies. Fasting blood samples were collected into vacutainer serum tubes (BD). Serum was collected after 15 min centrifugation at 3,200 rpm and 16°C and stored at -20°C until protein reading. Serum iron, ferritin, and haptoglobin levels were measured following standardized protocols from the Sérgio Franco Laboratory (Rio de Janeiro, Brazil).

### Tests of natural selection

To search for genomic footprints of natural selection in the *PKLR* region, several tests were performed on European (Italian, Iberian, Great Britain, Northern and Western European from Utah) and African (Nigerian, Kenyan, Gambian, Sierra Leonean) populations from the 1000 Genomes Project. We used the allele frequency differentiation method based on pairwise F_ST_ to identify instances of local adaptation (F_ST_ can range from 0 to 1). In general, positive selection tends to increase the degree of differentiation between populations beyond that expected by neutrality. F_ST_ analyses were performed by the Weir and Cockerham (1984) method implemented in VCFtools v. 0.1.15 in sliding windows (20bp steps by 5bp) to avoid spurious values for individual SNPs. Since rare variants can inflate F_ST_ estimates, variants with MAF<0.05 were excluded from this analysis, and the F_ST_ values per window were calculated using the “ratio of averages”, as suggested by Bhatia and colleagues (2013) [48]. A second class of tests was Tajima’s D that looks at the frequency spectrum of alleles to detect deviations from neutrality. A D<0 occurs when there is an excess of low frequency variants and can presume positive or purifying selection (or demographic signature of an expansion in population size) while D>0 indicates balancing selection (or demographic signature of a decrease in population size) [49,50]. Tajima’s D was calculated in VCFtools (no MAF cutoff was applied) considering several window sizes (100Kb, 75Kb, 50Kb and 25Kb) across the chromosome 1 and empirical *p* values were obtained by comparing the distribution of the Tajima’s D values. Lastly, statistics based on LD patterns across genomes were used to detect positive sweeps. Under neutral evolution, allele frequencies change randomly. In this case, a new variant will require many generations to reach a high frequency in the population. Conversely, in the scenario of positive selection, a rapid rise in frequency of a beneficial allele in few generations will preserve the original haplotype structure (high frequency haplotype with strong and long-range LD). Here, we used the Extended Haplotype Homozygosity (EHH) approach and correlated tests–Cross-population Extended Haplotype Homozygosity (xpEHH) and the Integrated Haplotype Score (iHS)–to detect signatures of recent selection. The analysis were performed using the R package “rehh” v.3.3.1 [51–53]. EHH measures compare the decay of homozigosity among haplotypes of individuals from the same population. iHS compares the EHH profiles between the derived allele and the ancestral allele in the same population and the xpEHH compares EHH among populations and underscores SNPs that are under selection in one population but not in another (scores higher than 2 indicates selection). The EHH analyses were performed with phased data. The definition of the ancestral alleles was obtained from Ensemble (http://ensembl.org/info/genome/compara/ancestral_sequences.html) and we kept only SNPs which this information was available. Finally, LD analysis was performed in HAPLOVIEW.

### Statistical analysis

Quality control (QC) was performed using PLINK 1.07 software [54]. The following criteria were applied: 1) Individuals with missing or discordant demographic information were removed (a total of 4,555 for the case-control studies remaining); 2) a genotyping call rate >90% per the entire sub-groups (cases or controls) was considered for the analysis and 3) SNPs were in agreement with the Hardy-Weinberg equilibrium (p<0.05). For the case-control studies, comparative allelic, genotypic, minor allele carriers and haplotype frequencies between groups were carried out using the logistic regression model as described previously [41,55]. The covariates “age”, “gender” and “genomic ancestry” were compared between cases and controls by the Chi-square (categorical variables) and Wilcoxon tests (numerical variables) in each population. Analyses were adjusted for the covariates significantly different between the groups to control for confounders. Deviations from Hardy-Weinberg equilibrium were tested using Chi-square for all control groups. Odds Ratio (OR) with 95% confidence interval was used to estimate the genetic association with leprosy or TB. Statistical tests were based on a two-tailed probability and the *p* values were adjusted for multiple comparisons (FDR<0.05). Logistic regression was performed using R software for Windows, using the packages “genetics” and “haplo.stats”. Meta-analysis was assessed by applying the generalized linear model with random effects to the “all Brazilians” single analysis by the R packages “arm” and “lmtest” using “region” as a random covariate to prevent false-positive associations due to population heterogeneity. The association tests were performed for the co-dominant (major allele homozygotes vs. heterozygotes / major allele homozygotes vs. minor allele homozygotes), dominant (major allele homozygotes vs. heterozygotes+minor allele homozygotes), recessive (major allele homozygotes + heterozygotes vs. minor allele homozygotes) and allelic models based in the test population (Rio de Janeiro). Haplotype frequency estimates were carried out using expectation–maximization (EM) algorithms. LD estimations were measured by r^2^ using the HAPLOVIEW software 4.2. For the functional comparisons, quantitative data were adjusted for covariates sex and/or age using least squares linear regression and graphics were plotted using “ggplot” v 3.2.1 in R. The linear model estimates the mean for each covariate and adjusts each observation towards this value. Median values of the serum measurements from each genotype group were compared by the two-tailed Mann-Whitney t-test (two groups of comparison) or Kruskal-Wallis test followed by Dunn’s post-test (three groups of comparison). Missing data was excluded from the analysis. *P values* of 0.05 were taken as statistically significant. Data from the Genotype-Tissue Expression Project (GTEx) were used to identify eQTLs SNPs from the website: https://gtexportal.org/home/ [56].

## Results

### *PKLR* SNPs are associated with leprosy susceptibility in Brazilian populations with higher African descent (Rio de Janeiro and Salvador)

**Fig 1:**
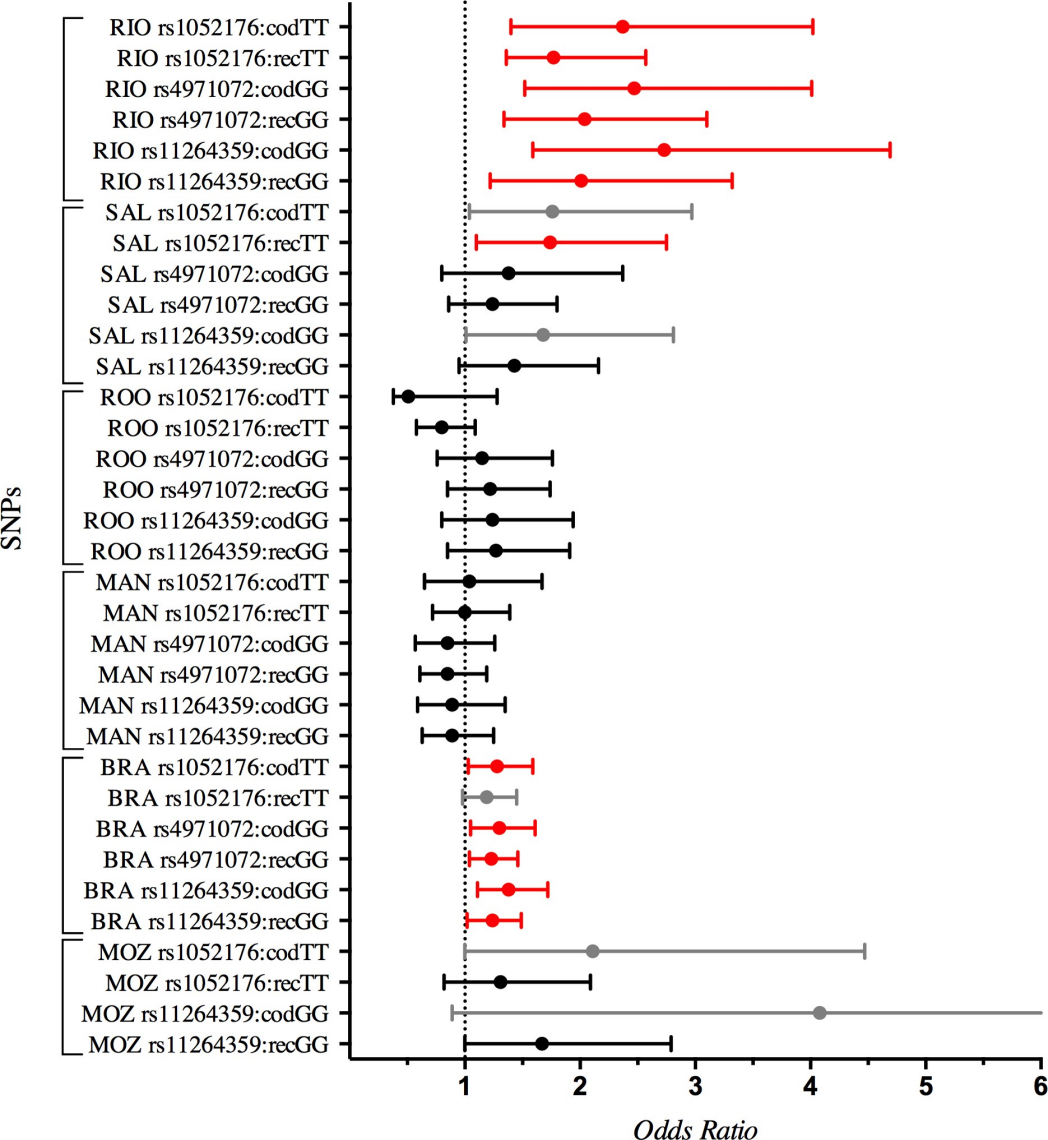
Genetic association of the *PKLR* SNPs with mycobacterial diseases in the studied population. Only the homozygous genotypes for the association, considered as “risk genotypes”, for the co-dominant (cod) and recessive (rec) models were represented for visualization purposes. The complete data of the individual SNPs are detailed in S3 Table. X-axis shows the Odds Ratio (OR) given in a linear scale. Red and gray dots with 95% confidence interval (CI) represented the significant (p<0.05) and *borderline* (0.05≤p≤0.07) associations, respectively. Black dots with 95% CI represented no association. RIO = Rio de Janeiro (Southeast Brazil); SAL = Salvador (Northeast Brazil); ROO = Rondonópolis (Midwest Brazil); MAN = Manaus (North Brazil); BRA = all Brazilians (meta-analysis) and MOZ = Mozambique (East Africa).

Following our candidate SNP selection methods, three tag SNPs (rs1052176, rs4971072 and rs11264359) covering the *PKLR* region were chosen for the association studies. Genotype frequency for the analyzed SNPs in the control group for each population or in the combined Brazilian analysis did not deviate from Hardy-Weinberg (HWE). In logistic regression, controlling for gender, age and ancestry and adjusting for FDR, the *PKLR* SNPs rs1052176, rs4971072 and rs11264359 were significantly associated with leprosy in Rio de Janeiro (S3 Table). As summarized in Fig 1, a susceptibility association with leprosy *per se* were observed for the genotype GT (ORcod_GT_ = 1.75, *p* = 0.003) and TT (ORcod_TT_ = 2.37, *p* = 0.001) in the co-dominant model, dominant (ORdom_GT/TT_ = 1.87, *p* = 0.0003) and recessive (ORrec_TT_ = 1.77, *p* = 0.02) models for rs1052176. SNP rs4971072 also presented a risk association for the GG genotype (ORcod_GG_ = 2.47, *p* = 0.0004) in the co-dominant model, as with the dominant (ORdom_AG/GG_ = 1.52, *p* = 0.02) and recessive (ORrec_GG_ = 2.04, *p* = 0.001) models, and following the same direction, genotype AG (ORcod_AG_ = 1.71, *p* = 0.003) and GG (ORcod_GG_ = 2.73, *p* = 0.0004) for the SNP rs11264359 were associated with leprosy in the co-dominant model, and the dominant (ORdom_AG/GG_ = 1.89, *p* = 0.0003) and recessive (ORrec_GG_ = 2.01, *p* = 0.006) models in Rio de Janeiro. The minor alleles of both the SNPs were also associated with leprosy in Rio de Janeiro (S3 Table).

As a replication approach, *PKLR* SNPs were tested in three populations from other Brazilian regions: Manaus (North), Salvador (Northeast) and Rondonópolis (Midwest). It was possible to observe a *borderline* signal for the *PKLR* association in Salvador for the TT genotype of rs1052176 (ORcod_TT_ = 1.76, *p* = 0.06) and GG genotype (ORcod_GG_ = 1.68, *p* = 0.06) in the co-dominant model and a significant association of rs1052176 in the recessive (ORrec_TT_ = 1.74, *p* = 0.03) model, while no replication was showed for the rs4971072. Salvador is the Brazilian population with the greatest African ancestry (SAL CO: 38% and SAL CA: 38%) in our study (S2 Table and S3 Fig). However, no association with leprosy was observed in Rondonópolis and Manaus (Fig 1 and S3 Table), which had the highest European (ROO CO: 59% and ROO CA: 58%—MAN CO: 36% and MAN CA: 36%) and Native-American (ROO CO: 16% and ROO CA: 14%—MAN CO: 38% and MAN CA: 35%) ancestries (S2 Table and S3 Fig), according to its historical composition.

To investigate the consistency of the association across the diverse Brazilian datasets, we considered all Brazilian populations as one (2,234 controls and 1,850 cases) and performed a meta-analysis employing random-effects model and adjusting for gender, age, ancestry and FDR. Then, we confirmed the susceptibility association of *PKLR* with leprosy for the genotypes TT-rs1052176 (ORcod_TT_ = 1.28, *p* = 0.02; ORrec_TT_ = 1.19, *p* = 0.06), GG-rs4971072 (ORcod_GG_ = 1.30, *p* = 0.01; ORrec_GG_ = 1.23, *p* = 0.03), and GG-rs11264359 (ORcod_GG_ = 1.38, *p* = 0.009; ORrec_GG_ = 1.24, *p* = 0.03) in the co-dominant and recessive models in Brazil with concise 95% confidence intervals (Fig 1 and S3 Table).

Linkage disequilibrium (LD) analysis showed that SNPs rs1052176, rs4971072 and rs11264359 are displayed in moderate LD in Rio de Janeiro and Salvador whereas Rondonópolis, and particularly Manaus, presented moderate-to-high LD between the markers (S4A–S4D Fig). In Mozambique, as with in Africans from the 1000 Genomes Project, these SNPs are not in LD (S4E and S4F Fig). It is reasonable that the different patterns of linkage disequilibrium between SNPs among the Brazilian populations may clarify the differences observed in our individual genetic association studies, explaining the inability to replicate the original findings from Rio de Janeiro in all populations. These results highlight the complexity of studying admixed multi-ethnic populations.

In the association study of Mozambique, the same trend was observed. The genotypes TT-rs1052176 (OR_TT_ = 2.11, *p* = 0.06) and GG-rs11264359 (OR_GG_ = 4.08, *p* = 0.06) presented a suggestive association to develop TB after the adjustments (Fig 1 and S3 Table). SNP rs4971072-Allele G exhibited 100% and 98% frequency in case and control groups, respectively, which suggests the maintenance of a high frequency allele in Africa, possibly due to a selective event. Altogether, these findings suggest the association of *PKLR* SNPs with susceptibility to leprosy *per se* in Brazil, particularly in populations with high African ancestry, like Rio de Janeiro and Salvador, and with TB in Mozambique.

### T/G/G (rs1052176/rs4971072/rs11264359) haplotype drives the susceptibility association with mycobacterial diseases in Brazilian and Mozambican populations

Haplotype analysis showed a significant susceptibility association with leprosy for the haplotype T/G/G (rs1052176/rs4971072/rs11264359) in Rio de Janeiro (OR = 1.64, *p* = 0.002), with an increased frequency in cases (0.36) compared to controls (0.28) (Table 1). Haplotype G/G/G was also associated with risk in Rio de Janeiro (OR = 2.23, *p* = 0.001), but it was not considered here due to its low frequency (<10%) among Brazilians. In Salvador, the Brazilian population with the greatest African ancestry, a high frequency of the haplotype T/G/G in cases (0.45) was also observed compared to controls (0.37), with a significant susceptibility association (OR = 1.54, *p* = 0.04) with leprosy. Association of the T/G/G haplotype with leprosy was also confirmed in the “all Brazilians” group (OR = 1.27, *p* = 0.02). Interestingly, the frequency of the haplotype T/G/G was higher in Africans (0.47) compared to Europeans (0.27) from the 1000 Genomes Project, as well as in Salvador (0.45) from the EPIGEN-Brazil Initiative compared to two Brazilian populations with higher European ancestry, Pelotas from the state of Rio Grande do Sul (South Brazil) (0.28) and Bambuí from the state of Minas Gerais (Southeast Brazil) (0.24) (S4 Table).

**Table 1:** Haplotype frequencies in the *PKLR* genomic region within populations and association with leprosy and TB.

SNPs			1000 Genomes		Leprosy															Tuberculosis		
**rs1052176**	**rs4971072**	**rs11264359**	**AFR**	**EUR**	**RIO CO**	**RIO CA**	**OR (CI)** ***P*** ^A^	**SAL CO**	**SAL CA**	**OR (CI)** ***P*** ^B^	**ROO CO**	**ROO CA**	**OR (CI)** ***P*** ^C^	**MAN CO**	**MAN CA**	**OR (CI)** ***P*** ^A^	**BRA CO**	**BRA CA**	**OR (CI)** ***P*** *	**MOZ CO**	**MOZ CA**	**OR (CI)** ***P*** ^B^
**T**	**G**	**G**	0.47	0.27	0.28	0.36	**2.46** (1.60–3.78) ***0*.*00001***	0.37	0.45	**1.57** (1.01–2.46) ***0*.*04***	0.33	0.32	0.77 (0.49–1.19) *0*.*24*	0.45	0.42	1.25 (0.79–1.97) *0*.*33*	0.37	0.38	**1.27** (1.04–1.57) ***0*.*01***	0.54	0.64	**1.52** (0.95–2.43) ***0*.*07***
**G**	**G**	**A**	0.32	0.04	0.09	0.07	ref	0.11	0.10	ref	0.06	0.09	ref	0.05	0.05	ref	0.07	0.08	ref	0.19	0.13	ref
**G**	**G**	**G**	0.19		0.04	0.08	**3.13** (1.54–6.39) ***0*.*001***	0.09	0.07	1.02 (0.56–1.85) *0*.*93*	0.04	0.06	1.14 (0.59–2.21) *0*.*68*				0.04	0.06	1.30 (0.95–1.76) *0*.*08*	0.23	0.20	1.26 (0.72–2.18) *0*.*40*
**G**	**A**	**A**		0.66	0.52	0.40	1.29 (0.86–1.95) 0.*21*	0.36	0.35	1.26 (0.81–1.97) *0*.*29*	0.52	0.49	0.76 (0.49–1.17) *0*.*22*	0.44	0.48	1.29 (0.81–2.05) *0*.*27*	0.46	0.43	1.07 (0.87–1.32) *0*.*48*			

Haplotypes with less than 5% of frequency in any of the groups were not shown. Bold *p* values are considered significant (*p*<0.05) or *borderline* (0.05≤p≤0.07). AFR = Africans (1000 Genomes Project); EUR = Europeans (1000 Genomes Project); RIO = Rio de Janeiro; SAL = Salvador; MOZ = Mozambique; ROO = Rondonópolis; MAN = Manaus; BRA = all Brazilians; CA = Cases; CO = Controls. Odds ratio (OR) and confidence intervals (CI) were calculated based on the reference (ref) haplotype.

^A^OR and *P*-values adjusted for covariates age, gender and ancestry.

^B^OR and *P*-values adjusted for gender and age.

^C^OR and *P*-values adjusted for ancestry.

*Meta-analysis of “all Brazilians” were performed by generalized linear model for random effects adjusted for gender, age and ancestry.

In the same way, 64% of the TB cases from Mozambique carry the haplotype T/G/G in contrast to 54% of the controls, and a susceptibility association with an indicative *p* value (OR = 1.52, *p* = 0.07) was seen in this population (Table 1). The difference in haplotype frequency was not observed among cases and controls from Manaus and Rondonópolis. Thus, these findings confirm the genetic relevance of the T/G/G *PKLR* haplotype on susceptibility to mycobacteria diseases in admixed populations exhibiting high-content African ancestry (Rio de Janeiro and Salvador) and African populations (Mozambique).

### Natural selection in the *PKLR* genomic region

In view of the large differences in the haplotype T/G/G frequencies between African and European populations (Table 1), we hypothesized that the *PKLR* genomic region could exhibit signatures of pathogen-driven selection. In order to identify possible selective signals, several tests were performed in the African and European populations obtained from the 1000 Genomes Project. Through population differentiation analyses (F_ST_), only the SNP rs4971072 was an *outlier* (F_ST_ = 0.66) for the genome-wide F_ST_ distribution with an empirical *p* value of 0.02 (S5 Fig and S5 Table). However, in face of a selective event, it is expected that the region neighboring the target SNP would exhibit the same selective signal, but the analysis of average within windows displaying the SNPs (rs4971072 and adjacent variants) of interest were not significant (*p*<0.05). Using Tajima’s D statistic calculated for windows of 100, 75, 50 and 25kb on the chromosome 1 in each population, we found no window providing significant evidence of selection for the *PKLR* region in Africans or Europeans (S6 Table).

In the EHH analyzes, despite the ancestral (allele G) and derived alleles (allele T) having slightly different patterns for the core SNP rs1052176, there was no clear sign of positive selection in Africans (S6 Fig). However, the comparison by the cross-population haplotype-based approach (xpEHH) between Africans and Europeans identified a candidate haplotype homozigosity region of 394 bp, with 25 SNPs (Table 2) (xpEHH > |-2.00|) at the gene *HCN3* in Europeans (S7A Fig). Interestingly, in both Africans and Europeans, a block in high LD was observed between 6 of the SNPs (rs12044063, rs7520184, rs11264352, rs11264353, rs11264354 and rs12724449) and the variants associated with susceptibility to leprosy and TB (Table 2 and S7B Fig). Furthermore, according to Genotype-Tissue Expression project (GTEx), these 6 SNPs are described as *PKLR* and *HCN3* expression quantitative trait loci (eQTLs), where the alternative allele of the variants downregulate the expression of those genes (Table 2). The xpEHH test looks for regions with extended haplotype homozigosity that differs between populations. Using this approach, we revealed evidence for selection favoring ancestral alleles in *HCN3* locus in Europeans. Interestingly, the region identified as positively selected in Europeans shows no evidence of recently favored variant in Africa, where alleles occur at an intermediate frequency. Taken together, our results have not directly confirmed evidence of recent positive selection of the *PKLR* in Africa, but raise the possibility that balancing selection plays a role in Africa, calling for further analysis (including specifically tests designed for balancing selection).

**Table 2:** Linkage disequilibrium between the SNPs associated with leprosy and the 25 SNPs with a suggestive selection sweep in European population by the cross-population xpEHH.

LD in AFR / EUR (1000 Genomes)						
*HCN3*	*PKLR*			eQTL	NES *PKLR* (*p* value)	NES *HCN3* (*p* value)
	rs1052176	rs4971072	rs11264359			
rs183293873	0 / *	0 / *	0 / *	No	-	-
rs12044063	**67 / 95**	0 **/ 73**	**62 / 92**	**Yes**	-0.58 (3.8x10^-**24**^)	-0.47 (6.4x10^-**52**^)
rs7367998	22 / *	1 / *	45 / *	No	-	-
rs560609863	0 / *	0 / *	0 / *	-	-	-
rs7520184	**88 / 97**	0 **/ 71**	**42 / 90**	**Yes**	-0.61 (3.8x10^-28^)	-0.47 (1.4x10^-54^)
rs11264352	**93 / 97**	0 **/ 71**	**46 / 89**	**Yes**	-0.61 (1.9x10^-27^)	-0.47 (2.5x10^-53^)
rs573791612	0 / *	0 / *	0 / *	-	-	-
rs541165488	0 / *	0 / *	0 / *	-	-	-
rs559902587	6 / *	0 / *	3 / *	No	-	-
rs11264353	**95 / 97**	0 **/ 71**	**45 / 90**	**Yes**	-0.61 (1.9x10^-27^)	-0.47 (2.5x10^-53^)
rs552421834	0 / *	0 / *	0 / *	-	-	-
rs564241242	0 / *	0 / *	0 / *	-	-	-
rs531296924	0 / *	0 / *	0 / *	-	-	-
rs549806272	0 / *	0 / *	0 / *	-	-	-
rs144733142	0 / *	0 / *	0 / *	No	-	-
rs535535805	0 / *	0 / *	0 / *	-	-	-
rs547542932	0 / *	0 / *	0 / *	-	-	-
rs11264354	**95 / 97**	0 **/ 71**	**45 / 90**	**Yes**	-0.61 (1.9x10^-27^)	-0.47 (2.5x10^-53^)
rs539661366	0 / *	0 / *	0 / *	-	-	-
rs558388981	0 / *	0 / *	0 / *	-	-	-
rs113065628	0 / *	0 / *	0 / *	No	-	-
rs144035252	8 / *	0 / *	3 / *	No	-	-
rs541106278	0 / *	0 / *	1 / *	No	-	-
rs559445057	0 / *	0 / *	0 / *	-	-	-
rs12724449	**43 / 99**	0 **/ 73**	**97 / 91**	**Yes**	-0.61 (5.6x10^-27^)	-0.47 (4.3x10^-52^)

*No pairwise LD data. Variant has a minor allele frequency close or equal to 0 in the population (Europeans). LD is represented in r^2^*100. Bold values are SNPs with moderate to high LD in Africans (Yoruba in Ibadan, Nigeria) and Europeans (Utah residents with European ancestry) of the 1000 Genomes Project, creating a large LD block with the SNPs associated with leprosy. The column of the eQTLs represents the SNPs that regulate the *PKLR* and *HCN3* expression in neural tissue by the GTEx Portal. The last column presents the Normalized Effect Size (NES) computed as the effect of the alternative allele relative to the reference allele in the human genome reference (hg19) and *p*-values.

Additionally, we addressed two findings for *PKLR* SNPs in a malaria dataset recently published by Gouveia and colleagues (2019) [12], which provides a comparison between an African population of intense malaria transmission (Ghana and Northern Uganda) and the South of Africa, where malaria is rare (Sotho and Zulu). SNP rs11264359 presented an F_ST_ of 0.079 between Ghana and Sotho/Zulu, which could be considered a substantial F_ST_ (25 times higher than the genome-wide F_ST_) whenever considering genetic distance among African populations (S7 Table). An xpEHH of 2.25 for SNP rs11264359 was highlighted among these populations, attesting that a suggestive recent positive selection signal was seen in Ghana, but not in Uganda, compared to Sotho and Zulu. However, a higher frequency of allele G for SNP rs11264359 occurred in Sotho and Zulu, not in Ghana as we had expected (S8 Table). We reasoned that allele G might have reached a higher frequency in Sotho and Zulu in the past timescale that malaria acted in this population.

### Risk *PKLR* variants and the levels of iron proteins and *PKLR* gene expression

We hypothesized that genetic variants selected by *Plasmodium* in Africa could impair RBC half-life leading to some perturbation in iron metabolism under the range of normality since healthy individuals do not experience any anemia. Thus, we tested whether *PKLR* variants were associated with alterations in circulating iron levels. Overall, serum iron was diminished in leprosy multibacillary (MB) cases compared to healthy subjects and paucibacillary (PB) cases, due to the chronic infection (S8 Fig). We also measured ferritin, which is widely used to test iron storage, and haptoglobin (Hp), the hemoglobin (Hb) scavenger in plasma, reducing the Hb toxicity by removal of the complex Hp-Hb through CD163 receptor [57–60]. Compared to healthy subjects, ferritin was decreased in PB and MB patients and haptoglobin was augmented in both PB and MB cases (S8 Fig).

**Fig 2:**
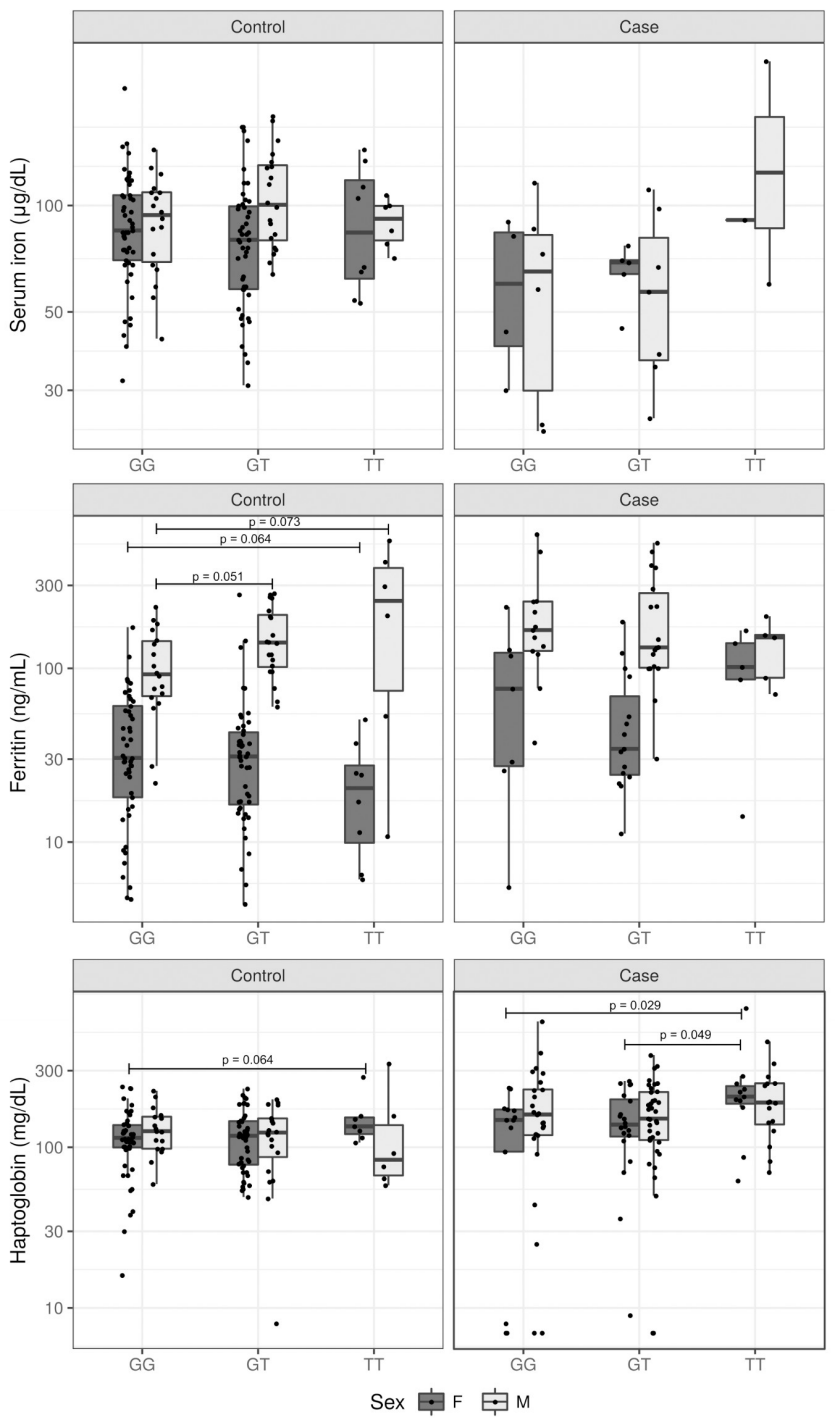
Serum iron, ferritin and haptoglobin levels among genotypes of the SNP rs1052176 by gender. Bars represent the median. Measurements in healthy subjects (Control) and leprosy patients (Case). Analysis were performed using Kruskal-Wallis test (*p*<0.05). F = Female; M = Male.

Compared with the genotype combination of SNPs rs1052176, rs4971072 and rs11264359 (T/G/G as the risk haplotype and G/A/A as the protection haplotype), no significant differences for iron and ferritin were found in healthy individuals or leprosy cases, but a suggestive increase (p = 0.07) of haptoglobin was observed for T/G/G compared to heterozygous haplotypes among cases (S9 Fig). Moreover, stratifying by gender, it was possible to observe subtle higher and diminished levels of ferritin for TT genotype of rs1052176 in healthy men and women, respectively (Fig 2). Furthermore, haptoglobin measurements were significantly increased in cases and healthy women carrying the TT genotype of the SNP rs1052176 (Fig 2). There were not enough samples available to consider the analysis of iron within this strategy.

We then retrieved genotype-phenotype correlations from public databanks and the T/G/G haplotype from *PKLR* SNPs were suggestive *PKLR* and also *HCN3* eQTLs in nerve tibial by GTEx (S10 Fig). There was a downregulation of *PKLR* expression in healthy individuals with the risk haplotype (represented by TT for rs1052176, GG for rs4971072 and GG for rs11264359). The effect of the eQTL variants on expression levels was higher in the tibial nerve, but they also present significant effect sizes in other tissues such as skin (not sun exposed–suprabubic), spleen and brain. Thus, together with the slight differences in the hematological parameters, it is possible that the risk haplotype could affect iron protein levels and the expression of *PKLR* or adjacent genes in a way that favors the infection.

## Discussion

Our study showed an association of putative malaria-resistance variants in the *PKLR* gene with susceptibility to leprosy and TB in Brazilian populations with higher African content (Rio de Janeiro and Salvador) and in Africa (Mozambique). Although the *PKLR* association with malaria outcome in human population remains to be confirmed, a large LD region among individuals with no malaria, and the association of one extended *PKLR* haplotype with mild malaria infection in Africa were reported, suggesting a conserved genomic region in this clinical group [22,24,25]. In this study, we explored how genetic footprints related to past selection events might be associated with disease susceptibility to leprosy and TB in admixed population, particularly those with higher African ancestry. This model helps explain why the replication did not occur in Brazilian populations with low African ancestry. In addition, as discussed in previous reports, it is hypothesized that selection across the *PKLR* genomic region could be shaped by *Plasmodium* exposure in Africa [21–25]. Our results supporting selection were suggestive, but insufficient to confirm this theory. We used approaches that detect selection signatures at different timescales. Tests of long-term selection (up to 200kya) (Tajima’s D) and intermediate timescales (75-25kya) (F_ST_) did not detect selection signals in the *PKLR* genomic region. On the other hand, tests for recent selection (up to 25kya) identified a signature of an extended haplotype homozigosity in a *PKLR/HCN3* region of eQTL SNPs for Europeans, but not for Africans (xpEHH test), where it was expected under the hypothesis of malaria-driven selection (see the summarized results in S9 Table). The *PKLR* association with mycobacteria in ethnic-specific Brazilian populations was observed after adjustments, but the evidence of selection in this dataset was complex, and further investigation of the microevolutionary forces on *PKLR* locus with additional population and larger sample sizes are necessary.

Admixed populations offer the opportunity for mapping disease-related variants with large allele frequency differences between ancestral populations. However, association studies in admixed cohorts need a rigorous control for genetic ancestry to avoid false-positive associations [61]. One interesting concern is that, although necessary, adjustments for genomic ancestry in admixed population may under-represent ancestry-dependent genetic associations. We were able to identify the *PKLR* susceptibility association solely in the two Brazilian populations with the historically highest African ancestry, Rio de Janeiro and Salvador, port cities where most of the slaves during 18^th^ century were admitted [30,38,62]. The populations of Rondonópolis and Manaus, conversely, carry greater European and Native American ancestry. Here, we suggested that the susceptibility association of the T/G/G haplotype with mycobacterial diseases in admixed population could be detected in populations with higher African content, meanwhile when lower rates of African ancestry are presented in a population, the genetic effect might be diluted. Even though the association signal was not strong in Salvador or in Mozambique (the number of individuals might be a limiting factor), it was confirmed when combining the Brazilian sites, corroborating the role of *PKLR* gene with leprosy within Brazil. Additionally, in agreement with our hypothesis, the association of the SNPs rs1052176 (Allele G–OR = 0.78, *p* = 0.01), rs11264359 (Allele G–OR = 0.82, *p* = 0.06), and rs4971072 (Allele G–OR = 0.81, *p* = 0.04), with leprosy *per se* was also corroborated in the Han Chinese genome-wide association study (GWAS) from Zhang and colleagues (2009) (S10 Table) [63]. Although opposite OR were observed for the risk alleles in Rio de Janeiro due to differences in Asian genomic architecture, this result reinforces our hypothesis and underscores some relevance of the *PKLR* gene with susceptibility to leprosy.

African descent populations may carry selective footprints of malaria resistance, as already seen for HbS in *quilombos*, settlements of people of African origin in Brazil [64]. Here, though analysis regarding the association to malaria specifically is required, this hypothesis was endorsed due to the African inheritance of Brazilians [65] and the high frequency of the risk haplotype among leprosy and TB cases, as well as Africans from the 1000 Genomes Project and Salvador from the EPIGEN-Brazil Initiative. To examine whether *PKLR* is under selection we applied tests that were designed to identify positive selection, in particular hard sweeps, which may not be the type of selection acting on *PKLR* genomic region. We speculate that balancing selection, which maintains alleles in the population over long evolutionary timescales, should be further investigated as a mechanisms for selection in the *PKLR*. Berghout and colleagues (2012) have also indicated some evidence of selection in the *PKLR* region by Tajima’s D, Fu and Li’s D and F [24]. Several other genes with genetic variants associated with resistance to malaria (e.g. *HBB*, *ABO*) are under balancing selection [66]. This selective regime would also explain the absence of signal by the EHH approach. On the other hand, an EHH candidate region was observed by the xpEHH approach in Europeans, which have intermediate allele frequencies in Africans, including 6 SNPs described as *PKLR* and *HCN3* eQTLs. The *HCN3* gene, adjacent to *PKLR*, encodes for a voltage-gated channel performing ionic, potassium and sodium transport highly expressed in early erythroid cells. Evidence for association between the *HCN3* variants and malaria in Africa have been raised by Machado and colleagues (2010), who observed a conserved haplotype and high heterozygosity at this gene associated with an uncomplicated malaria group in Angola [25]. Consistently, variants at *HCN3* have been associated with RBC deficiency and the variant rs1052176 has been linked with malaria [23,25,67]. Moreover, variants under selection in the *HCN3* are in LD with the SNP associated with leprosy susceptibility–rs1052176 –which is highlighted as an eQTL for *PKLR* and *HCN3* genes by GTEx. Additionally, an extreme xpEHH (+2.25) was detected for the SNP rs11264359 between Ghana and Sotho/Zulu, although the most frequent allele in Ghana for rs11264359 (and rs1052176) was not the risk G allele associated with leprosy and it is likely that some other geographic factors might be involved in this scenario. Overall, given the absence of compelling signals throughout the tests, we could not confirm that *PKLR* region is subject to natural selection in our dataset. Thus, our exploratory data gives direction, but the scenario of *PKLR* selection is much more complex.

Due to its involvement with ATP production and erythrocyte integrity, the *PKLR* gene is a putative target of selection being associated with adaptation to high-altitude in Tibetans and with malaria in Sub-Saharan Africa [25,68,69]. Hemolysis releases heme, which is detoxified inside macrophages in iron and other metabolites. Iron overload, observed among some PK-deficient patients [70], might favor intracellular pathogens and mycobacteria infection [27,71,72]. Here, despite the evidence of association observed in our studies, a mild functional correlation with the risk haplotype of the *PKLR* gene and iron measurements was detected. As expected, we observed an increase in ferritin levels in TT-genotype men (rs1052176), but not in women. Importantly, iron levels are affected by gender and other factors, such as diet, exercise and other genes that should be considered for a better correlation of the *PKLR* genotypes and iron levels, but it is outside the scope of this study [73]. At the molecular level, pyruvate kinase seems to be highly sensitive to even small changes. Computational tools predicted that the SNP rs1052176 may affect translation [67]. In addition, the variant rs4971072 is located in binding sites of transcriptional factors and may reduce PK levels, which might lead to malaria resistance [74]. However, an increase in reticulocytes or an overexpression of the *PKLR* gene may overcome possible functional damages on the levels of the enzyme [75]. Notwithstanding, data from GTEx showed that the haplotype T/G/G decreases the *PKLR* expression in nerve, and once it is confirmed for RBCs, it might alter the erythrocyte lifespan, consistent with our hypothesis.

**Fig 3:**
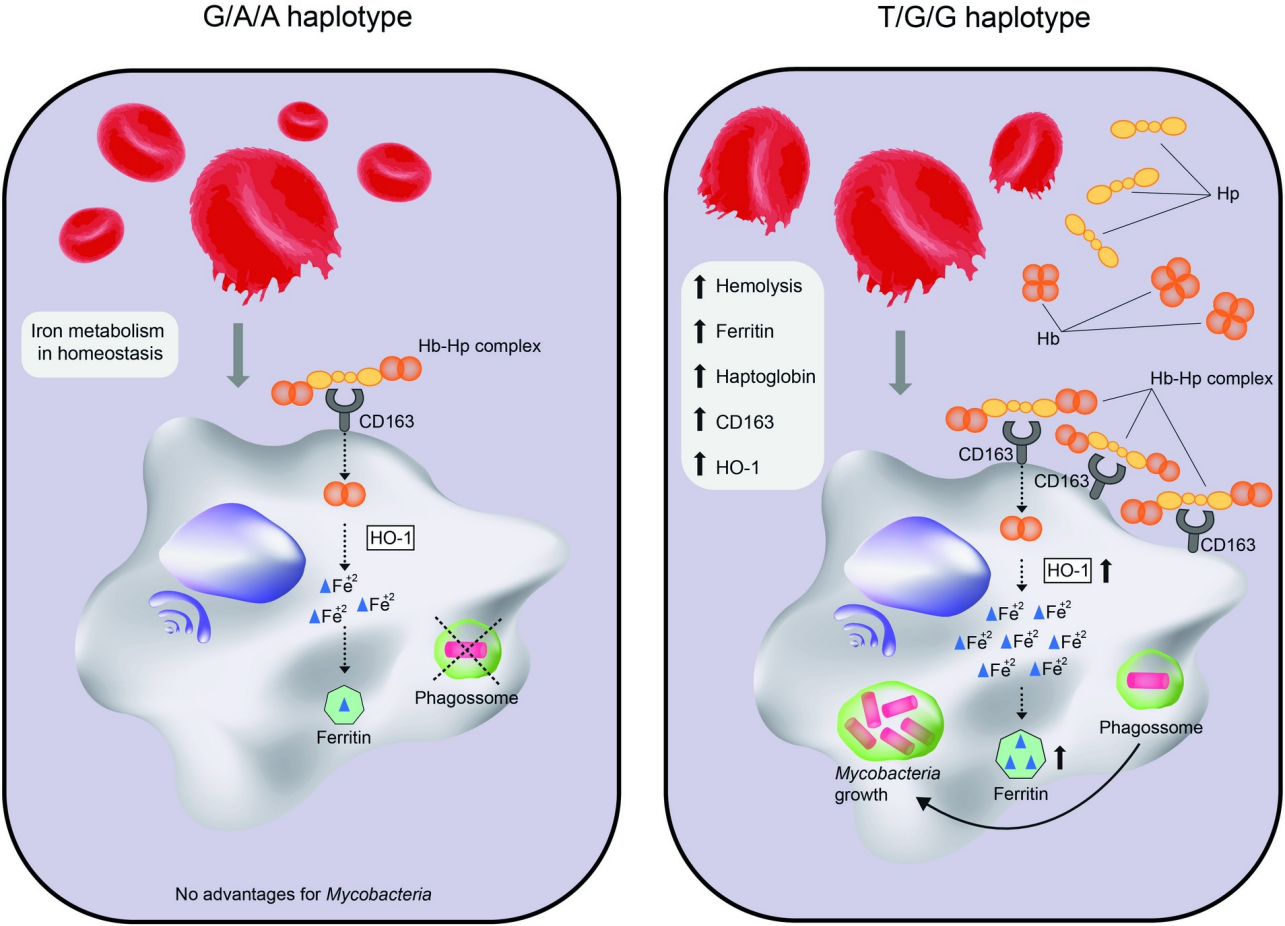
Putative mechanism of mycobacterial infection in the presence of the *PKLR* risk haplotype. The scheme illustrates the possible pathway that led to increase in ferritin levels and consequently *M*. *leprae* persistence inside macrophages. We hypothesized that the RBCs of T/G/G individuals could speed the hemolysis rate of erythrocytes with low levels of PK. Because of the hemoglobin (Hb) release, haptoglobin (Hp) is increased and the complex Hp-Hb internalizes heme inside macrophages via CD163. Heme-oxigenase 1 (HO-1) cleaves heme into iron, which is stored inside ferritin. Hemolysis is a common mechanism that occur in homeostasis to prevent oxidative stress. However, it can be enhanced in the presence of genetic determinants (*PKLR* T/G/G haplotype), leading to a subtle iron overload and a possible advantage for mycobacterial growth.

Our previous data showed that Hb, Hp, CD163 and iron deposits are augmented in skin lesions from multibacillary (MB) patients, which induces anti-inflammatory pathways favoring *M*. *leprae* survival [76,77]. Heme uptake is employed by *Mycobacterium tuberculosis* as a source for iron acquirement [78]. Internalization by the Hp-Hb complex may be an alternative strategy for iron uptake in *M*. *leprae* [79–81] and would explain the *PKLR* susceptibility association, as summarized in Fig 3. It is important to mention that the PK-deficiency is a very heterogeneous condition and the presence of causal mutations will not necessary reproduce the biological cost [82]. Additionally, it is likely that the cellular microenvironment might be more informative than serum measurements to better detect the slight genotype-phenotype meaning defended here.

Previously, a method based on the PCA for the identification of LD-groups and SNPs representing the diversity of a single genomic region was performed [34]. Here, by using the PCA as a complementary approach for the SNPs selection, we captured variants in the *PKLR* region that vary in frequency among populations and are in LD with the long-range haplotype under selection by the xpEHH. The “top SNPs” had the stronger influence in clustering the three ancestries in the PC1, which captured most of the intragenic variation. PC2, in turn, did not display higher “weights” for the same variants. However, it is reasonable that other PCs might also indicate functional variants and we emphasize considering other PCs when testing alternative genes. Notably, the use of PCA, which has performed well here, warrants further exploration. The PCA highlighted SNPs with a degree of linkage disequilibrium. After the LD pruning based in the African plot, we observed the association signal for three variants tagging distinct blocks and arranged in an extended and conserved haplotype. While the associated variants play a role in *PKLR/HCN3* expression in nerve and skin, the association signal might also arise from other variants in LD with the selected variants or even in adjacent genes not covered by our analysis.

Pathogen-driven selection implies that effective advantageous alleles against the pathogen oscillate (depending on the type of selection) across populations in endemic areas [83,84]. Taken together, we discuss the idea of a trade-off mechanism, in which the genetic association of *PKLR* variants, possibly selected for their malaria resistance in Africa, is also associated with susceptibility to mycobacterial diseases and, because of the heterogeneity in the Brazilian population, could be possible to detect selection signatures in the *PKLR* region among African-descendent populations. The trade-off mechanism might occur in other central genes of immune response and biochemical pathways, controlling the susceptibility to infections [85,86]. In general, GWAS in neglected diseases considering admixed individuals represent a small part of the large-scale genomic analysis. Studies in admixed populations increase diversity in genomics and could enhance our ability to understand the genetic architecture of human diseases [87,88]. Until now, there have been no studies investigating the association of energetic metabolism genes with mycobacterial diseases susceptibility, although the energetic metabolism seems to play central role during *M*. *leprae* infection [89,90]. As leprosy and TB are controlled by multiple variants (with “major” or “minor effect” in disease) and large-scale GWAS for disease traits predominantly identify common variants with weak effects (OR~1.2–1.7) shared across populations, the *PKLR* gene seems to contribute to this panel [91,92]. Finally, our study brings new insights into the complexity of how past selection events may influence present-day host susceptibility to infectious diseases, such as leprosy and TB.

## Supporting information

S1 Appendix SNPs selection methodology. (DOCX)

S1 Fig Selection strategy of the candidate SNPs. **A)** Diagram including the steps for the SNPs selection. From the initial variants in the PCA analysis, 30 “top SNPs” were selected and compared with the allele frequency, linkage disequilibrium (LD) and haplotype analysis, functional annotation, and literature reports. The Epigen Consortium was assessed to observe the frequency of the variants and haplotypes among the Brazilian samples. From the 3 candidate SNPs, only the rs11264359 did not match the ANNOVAR criteria (dashed line). **B)** Principal Component Analysis (PCA) of the *PKLR* SNPs in the populations of the 1000 Genomes Project. We used variants from a region of 10,000 bp *upstream* and *downstream* of the gene *loci* (chr1:155,259,084–155,271,225 –GRCh37/hg19) to observe the clusters displayed by each Principal Component (PC). Then, we evaluated the SNPs with the 30 highest scores (“SNP weight”) for the PC1 and, comparatively with the other analysis, we selected three candidate SNPs, given in S1 Table. EUR: Europeans; AFR: Africans and AMR: Native-Americans.
(TIFF)

S2 Fig LD map of the 30 “top SNPs” in Africans and Europeans from the 1000Genomes Project. LD plot (r^2^*100) of the 30 top variants of the *PKLR* gene covering representative blocks of the region in the 1000 Genomes populations. In red, we observe the blocks represented by the tag SNPs detached in Africans and, in yellow, we highlighted the conserved block in high LD under selection by the xpEHH.
(TIFF)

S3 Fig Proportions of the genetic ancestry of cases and controls from each population of the study. Parental populations from the HGDP-CEPH are represented in the blue (Native-American ancestry), red (African ancestry) and green bars (European ancestry). CO = Controls; CA = Cases; RIO = Rio de Janeiro; SAL = Salvador; ROO = Rondonópolis; MAN = Manaus and MOZ = Mozambique.
(TIFF)

S4 Fig Linkage disequilibrium among *PKLR* SNPs in the populations of the study. LD were calculated in r^2^. **A-E)** First and second LD plots in each population represents LD in controls and patients, respectively. **F)** LD in healthy European and African individuals from the 1000 Genomes Project.
(TIF)

S5 Fig Pairwise F_ST_ analysis in the 1000 Genomes populations. F_ST_ of 60 SNPs range in the *PKLR* genomic region between Africans (AFR) and Europeans (EUR) highlighting the F_ST_ and the empirical *p* values of the SNPs associated with mycobacteria. The dashed line represents the 95% quantile of the F_ST_ distribution along the chromosome 1. Red: rs1052176; blue: rs4971072; green: rs11264359.
(TIFF)

S6 Fig **Decay of haplotypes (EHH) from the SNP core in Africans (A) and Europeans (B) from the 1000 Genomes Project.** Horizontal lines are haplotypes, SNP positions are marked by the x-axis and the core SNP (rs1052176) position is represented by the dotted line. Blue indicates the EHH decay of the ancestral allele and red indicates the EHH decay of the derived allele. When the core SNP is neutral, the haplotype homozygosity decays at similar rates for both ancestral and derived alleles. When the derived alleles are favored, the haplotype homozygosity decays much slower for the derived alleles than for the ancestral alleles.
(TIFF)

S7 Fig Selection sweep by xpEHH (1:155247308–155290457) between African and Europeans in the *HCN3* gene and linkage disequilibrium between the SNPs. **A)** The signal (>|2.00|) of a sweep was observed for 25 SNPs in the *HCN3* gene across 1060 variants covering *HCN3*, *PKLR* and *FDPS* genes in Europeans. **B)** Linkage disequilibrium (r^2^*100) between the SNPs within the *HCN3*, *PKLR* and *FDPS* genes in Europeans and Africans from the 1000 Genomes Project.
(TIF)

S8 Fig Serum iron, ferritin and haptoglobin levels among individuals of the study. Bars represent the median of serum protein levels in each group adjusted by gender. Analysis were performed using Kruskal-Wallis test (*p*<0.05). Control = Healthy individuals; PB = Paucibacillary leprosy patients; MB = Multibacillary leprosy patients and ns = non-significant.
(TIF)

S9 Fig Serum iron, ferritin and haptoglobin levels for different *PKLR* haplotypes. Bars represent the median adjusted by gender. G/A/A = Protection haplotype; Hetero. = Haplotype of the heterozygous and T/G/G = Risk haplotype from heterozygous individuals for the SNPs rs1052176, rs4971072 and rs11264359. Analysis were performed using Kruskal-Wallis test (*p*<0.05). **A)** Measurements in healthy subjects and **B)** Measurements among leprosy cases.
(TIF)

S10 Fig *PKLR* gene expression by genotypes of the eQTLs SNPs. Median of the *PKLR* expression in the violin plot according to the genotype of each SNP. Number of individuals is represented in parentheses. Data were obtained of nerve biopsies according to the GTEx project, where the SNPs are eQTLs for the *PKLR* (rs1052176 *p* = 4.5x10^-29^, rs4971072 *p* = 2.9x10^-19^ and rs11264359 *p* = 1.1x10^-22^) and *HCN3* (rs1052176 *p* = 1.4x10^-53^, rs4971072 *p* = 2.1x10^-40^ and rs11264359 *p* = 3.3x10^-48^) genes and with minor significance for the *GBAP1* (rs1052176 *p* = 6.2x^-10^, rs4971072 *p* = 1.8x10^-10^ and rs11264359 *p* = 2.2x10^-10^), *RIT* (rs1052176 *p* = 4.0x10^-19^, rs4971072 *p* = 1.9x10^-12^ and rs11264359 *p* = 4.7x10^-11^) and *FAM189B* (rs1052176 *p* = 4.0x10^-5^, rs4971072 *p* = 1.1x10^-6^ and rs11264359 *p* = 1.8x10^-5^) genes.
(TIF)

S1 Table *PKLR* SNPs selected by functional annotation, allele frequency, linkage disequilibrium (LD), PCA analysis and literature reports. (XLSX)

S2 Table Characteristics of the populations enrolled in the study. (XLSX)

S3 Table Frequency of the *PKLR* SNPs in cases and controls from each tested population and logistic regression for association with leprosy or tuberculosis. (XLSX)

S4 Table Haplotype frequencies in the *PKLR* genomic region within populations of the 1000 Genomes Project and EPIGEN-Brazil. (XLSX)

S5 Table Analysis of FST, iHS and xpEHH of *PKLR* SNPs among Europeans and Africans from the 1000 Genomes Project. (XLSX)

S6 Table Tajima’s D for the windows containing the SNPs associated with mycobacteria. (XLSX)

S7 Table Analysis of FST, iHS and xpEHH for *PKLR* SNPs among African populations from the study of Gouveia et al. (2019) [12]. (XLSX)

S8 Table Allele frequency of the *PKLR* SNPs in Gouveia et al. (2019) [12] and in the 1000 Genomes Project populations. (XLSX)

S9 Table Summary of the principal findings observed for each of the *PKLR* variants in the study. (XLSX)

S10 Table *PKLR* association with leprosy in Han Chinese GWAS from Zhang et al. (2009) [63]. (XLSX)
