# Supplementary material for: Putative pathogen-selected polymorphisms in the PKLR gene are associated with mycobacterial susceptibility in Brazilian and African populations
Source: PLoS Negl Trop Dis. 2021 Aug 27;15(8):e0009434. doi: 10.1371/journal.pntd.0009434 (PMC8396769; doi:10.1371/journal.pntd.0009434)
Supplement: S1 Appendix — (DOCX) [file pntd.0009434.s001.docx]

**Supporting Information**

**S1 Appendix – SNPs selection methodology**

To search for possible functional candidate SNPs in the *PKLR* region we used data from the public database of 1000 Genomes Project phase III (504 Africans, 503 Europeans and 347 Native Americans) [1] and the EPIGEN-Brazil project (90 from Salvador, 88 from Bambuí and 87 from Pelotas) [2–4]. Thus, the allele and haplotype frequencies could be evaluated in the admixed Brazilians’ ancestral populations and in three Brazilian cohorts from different regions of the country (Northeast, Southeast and South). Then, we performed the analysis below:

1) We downloaded all the chromosome 1 variants of the 1000 Genomes (GRCh37/hg19) and filtered for the polymorphisms in the *PKLR* and flanking regions ~10,000bp *downstream* and *upstream* of the gene (155,247,084bp to 155,283,225bp) in the populations of interest, excluding the admixed population (ASW and ACB). The allele frequencies of the variants were calculated using PLINK [5];

2) The variants (n=921) were analyzed by the Principal Component Analysis (PCA) through EIGENSOFT [6,7], which were the starting point to select the candidate SNPs (S1A Fig). In the PCA, all data variability is reduced to the smallest possible number of principal components (PC) capable of recovering the maximum of the dataset variability. In other words, a large set of variables are collapsed into a smaller one that still represents most of the information of the large set. Each PC is composed of a linear combination of all the variables involved in the analysis with different “weights” assigned to each one. The “weights” represent how much each of these variables is responsible for explaining the variability recovered by each PC. Here, we indexed each individual’s genotype data of Africans, Native-Americans and Europeans and evaluate the clusters displayed in each PC-plot. Since each PC represents the combination of the genotype values for the *PKLR* variants, those capturing the higher percentages of variability in the analysis represent the variants where the genotypic frequencies most vary between the clusters, probably due to genetic drift or evolutionary forces. Whenever using genome-wide variants in where the key factors influencing variability are, in fact, the demographic factors in Africans or Europeans, the top SNPs with higher “weights” are influenced by ancestry. On the contrary, in this study, as we are looking for a specific gene in the genome, we hypothesized that the variability observed in the PC-plot could be ruled by the gene functionality. Thus, we assumed that the SNPs with higher “weights” in the PC1 (or PC2) might play a functional role at the gene possibly targeted by natural selection, which might lead to a phenotypic variation in the protein. Here, we have focused in the PC1 because it captured most of the intragenic diversity after first evaluation and pinpointed variants with previous biological relevance at the gene/protein in the literature, but PC2 or other PCs should also be informative when looking for other genomic regions. The PCA approach was supported by the hypothesis that *PKLR* is under natural selection, and interestingly, we could selected SNPs previously highlighted in PK deficiency and malaria studies, but the method as implemented here might not be a rule for other candidate genes. We then selected the 30 SNPs with the highest “SNP weights”, which we called “30 top SNPs”, and were the start point of our filtering. So, the 30 top SNPs were compared with the allelic frequency calculated in step 1 (keeping the SNPs with MAF>10% in Europeans and Africans), LD analysis, functional category and literature, as described. The following analyzes were comparatively used to refine the candidates SNPs;

3) Linkage disequilibrium (LD) analysis and haplotype frequencies were measured by HAPLOVIEW [8] to refine the selection of tag SNPs representative of the genomic region. SNPs with r²>0.80 was considered as in high LD and were pruned in the analysis. We kept SNPs tagging bins and that were in low to moderate LD with them, based in the African plot (since there was a high LD in Europeans) (S2 Fig). SNPs in a block of high LD were selected based on the genomic annotation (next step) to choose the tag of the bin taken into account those in coding/regulatory regions. Thus, SNPs from different bins arranged to form haplotypes were selected to cover an extended genomic region. Then, the haplotype analysis was also evaluated to search for the variant guiding the haplotype frequency differentiation between Africans and Europeans;

4) SNPs were annotated by ANNOVAR [9] with refGene hg19 (11 Dec 2015). They were classified as exonic (synonymous or not synonymous), intergenic, upstream, downstream, 5'UTR, 3’UTR or intronic. Annotation was used to select from the SNPs in high LD and/or located in coding or regulatory regions;

5) Finally, the variants were compared with the literature, and, as inclusion criteria, we kept the variants which were already highlighted in PK deficiency and malaria related studies [10–13]. In the table below, we show the “30 top SNPs” selected by the PCA and, in bold, the tag SNPs defined as candidate SNPs for the case-control association studies after the comparison with the further analysis.

**The 30 top SNPs selected by the PCA**

| **Nº** | **SNP** | **SNP weight (PC1)** | **Allele 1** | **Allele 2** | **EUR** | **AFR** | **Annotation** |
| --- | --- | --- | --- | --- | --- | --- | --- |
| 1 | rs12724449 | 4.141 | C | A | 0.282 | 0.666 | intronic |
| 2 | rs12741350 | 4.141 | T | C | 0.282 | 0.607 | intronic |
| 3 | rs11264357 | 4.140 | T | C | 0.282 | 0.282 | intronic |
| 4 | rs7554780 | 4.135 | T | G | 0.279 | 0.614 | intronic |
| 5 | rs2071053 | 4.134 | G | A | 0.283 | 0.621 | intronic |
| 6 | rs3020781 | 4.134 | G | A | 0.282 | 0.606 | intronic |
| 7 | rs7543234 | 4.132 | T | C | 0.279 | 0.663 | intronic |
| 8 | rs6672284 | 4.124 | T | C | 0.277 | 0.667 | upstream |
| 9 | rs12067675 | 4.093 | C | T | 0.282 | 0.657 | intronic |
| 10 | rs7534795 | 4.088 | T | C | 0.268 | 0.667 | intergenic |
| 11 | rs12049375 | 4.087 | A | G | 0.279 | 0.563 | intronic |
| **12** | **rs11264359** | **4.085** | **G** | **A** | **0.281** | **0.719** | **intronic** |
| 13 | rs4620533 | 4.083 | G | C | 0.278 | 0.447 | intronic |
| 14 | rs12044063 | 4.082 | T | A | 0.283 | 0.563 | intronic |
| 15 | rs3814319 | 3.910 | A | G | 0.330 | 0.670 | UTR3 |
| 16 | rs7549276 | 3.847 | A | G | 0.279 | 0.721 | intronic |
| 17 | rs11264355 | 3.798 | G | C | 0.281 | 0.474 | intronic |
| 18 | rs932972 | 3.788 | A | G | 0.280 | 0.472 | UTR3 |
| 19 | rs8847 | 3.786 | T | C | 0.280 | 0.471 | UTR3 |
| 20 | rs3814318 | 3.785 | T | C | 0.280 | 0.471 | UTR3 |
| **21** | **rs1052176** | **3.784** | **T** | **G** | **0.280** | **0.473** | **exonic (synonymous)** |
| 22 | rs1052177 | 3.784 | G | A | 0.280 | 0.473 | UTR3 |
| 23 | rs11264353 | 3.779 | C | G | 0.279 | 0.479 | intronic |
| 24 | rs11264354 | 3.779 | G | A | 0.279 | 0.479 | intronic |
| 25 | rs11264352 | 3.751 | C | T | 0.280 | 0.484 | intronic |
| 26 | rs8177968 | 3.716 | T | C | 0.282 | 0.458 | intronic |
| 27 | rs7520184 | 3.696 | A | G | 0.279 | 0.463 | intronic |
| 28 | rs7524950 | 3.630 | T | C | 0.282 | 0.440 | intergenic |
| **29** | **rs4971072** | **3.535** | **G** | **A** | **0.327** | **0.996** | **intergenic** |
| 30 | rs12032720 | 3.305 | C | G | 0.277 | 0.373 | intronic |

Allele frequencies refer to Allele 1. Descending values of “SNP weight” represented by the PC1 (Principal Component 1). EUR=Europeans and AFR=Africans from the 100Genomes. Annotation was performed by ANNOVAR3 (refGene hg19). In red are highlighted the variants under selection by the xpEHH which are in a LD bin with the associated variants.

**References**

1. The 1000 Genomes Project Consortium. A global reference for human genetic variation. Nat Author Manuscr. 2015;526: 68–74. doi:10.1038/nature15393.A

2. Victora CG, Barros FC. Cohort profile: The 1982 Pelotas (Brazil) birth cohort study. Int J Epidemiol. 2006;35: 237–242. doi:10.1093/ije/dyi290

3. Lima-Costa MF, Firmo JOA, Uchoa E. Cohort profile: The Bambuí (Brazil) cohort study of ageing. Int J Epidemiol. 2011;40: 862–867. doi:10.1093/ije/dyq143

4. Barreto ML, Cunha SS, Alcântara-Neves N, Carvalho LP, Cruz ÁA, Stein RT, et al. Risk factors and immunological pathways for asthma and other allergic diseases in children: Background and methodology of a longitudinal study in a large urban center in Northeastern Brazil (Salvador-SCAALA study). BMC Pulm Med. 2006;6. doi:10.1186/1471-2466-6-15

5. Purcell S, Neale B, Todd-Brown K, Thomas L, Ferreira MAR, Bender D, et al. PLINK: A tool set for whole-genome association and population-based linkage analyses. Am J Hum Genet. 2007;81: 559–575. doi:10.1086/519795

6. Price AL, Patterson NJ, Plenge RM, Weinblatt ME, Shadick NA, Reich D. Principal components analysis corrects for stratification in genome-wide association studies. Nat Genet. 2006;38: 904–909. doi:10.1038/ng1847

7. Horne BD, Camp NJ. Principal Component Analysis for Selection of Optimal SNP-Sets That Capture Intragenic Genetic Variation. Genet Epidemiol. 2004;26: 11–21. doi:10.1002/gepi.10292

8. Barrett JC, Fry B, Maller J, Daly MJ. Haploview: Analysis and visualization of LD and haplotype maps. Bioinformatics. 2005;21: 263–265. doi:10.1093/bioinformatics/bth457

9. Wang K, Li M, Hakonarson H. ANNOVAR: Functional annotation of genetic variants from high-throughput sequencing data. Nucleic Acids Res. 2010;38: 1–7. doi:10.1093/nar/gkq603

10. Machado P, Pereira R, Rocha AM, Manco L, Fernandes N, Miranda J, et al. Malaria: Looking for selection signatures in the human PKLR gene region. Br J Haematol. 2010;149: 775–784. doi:10.1111/j.1365-2141.2010.08165.x

11. Van Bruggen R, Gualtieri C, Iliescu A, Cheepsunthorn CL, Mungkalasut P, Trape JF, et al. Modulation of malaria phenotypes by pyruvate kinase (pklr) variants in a Thai population. PLoS One. 2015;10: 1–18. doi:10.1371/journal.pone.0144555

12. Berghout J, Higgins S, Loucoubar C, Sakuntabhai A, Kain KC, Gros P. Genetic diversity in human erythrocyte pyruvate kinase. Genes Immun. 2012;13: 98–102. doi:10.1038/gene.2011.54

13. Qidwai T, Jamal F, Singh S. Exploring putative molecular mechanisms of human pyruvate kinase enzyme deficiency and its role in resistance against Plasmodium falciparum malaria. Interdiscip Sci Comput Life Sci. 2014;6: 158–166. doi:10.1007/s12539-013-0025-8
