# Supplementary figures and images for: Putative pathogen-selected polymorphisms in the PKLR gene are associated with mycobacterial susceptibility in Brazilian and African populations
Source: PLoS Negl Trop Dis. 2021 Aug 27;15(8):e0009434. doi: 10.1371/journal.pntd.0009434 (PMC8396769; doi:10.1371/journal.pntd.0009434)

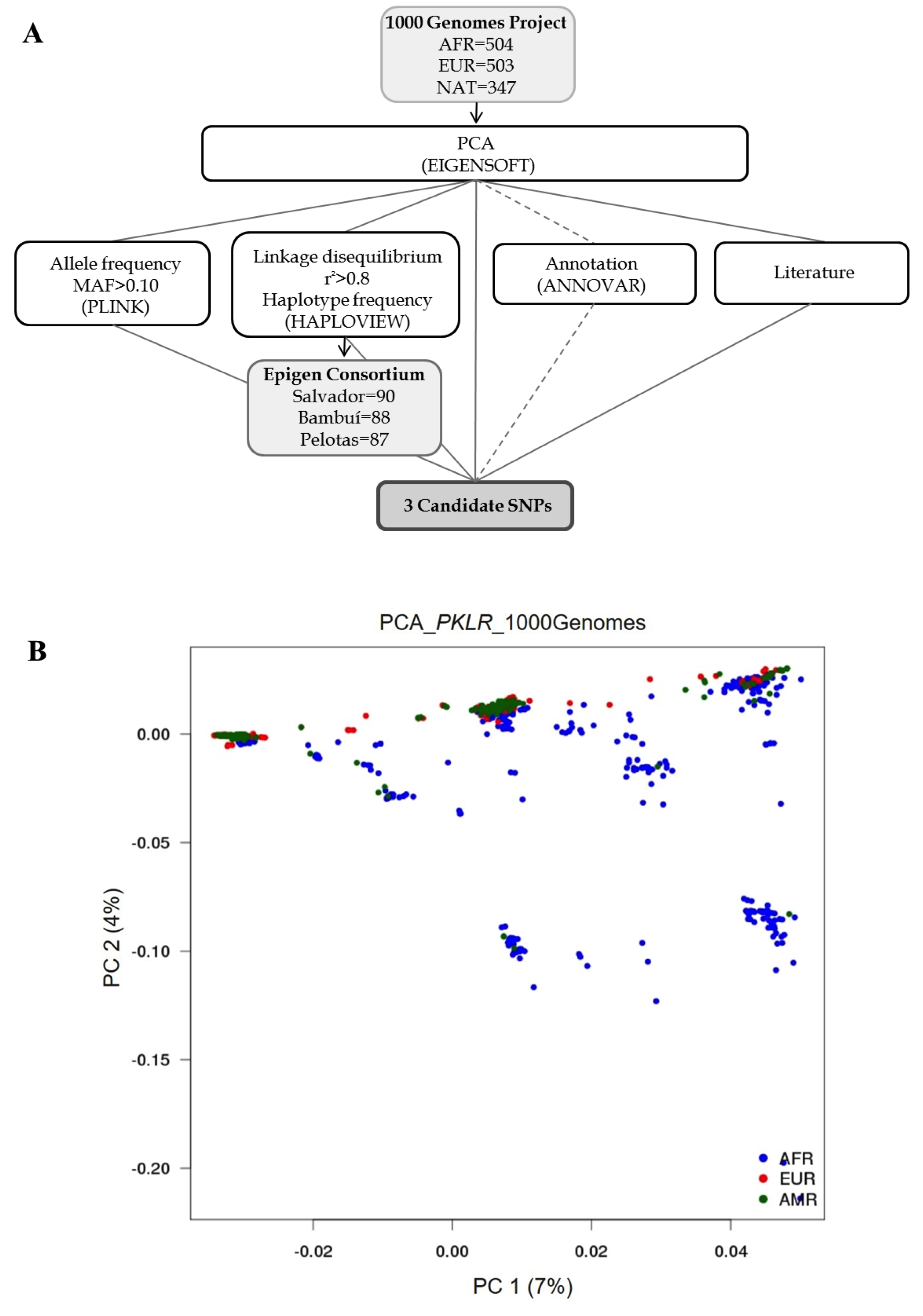

Supplement: S1 Fig — A) Diagram including the steps for the SNPs selection. From the initial variants in the PCA analysis, 30 “top SNPs” were selected and compared with the allele frequency, linkage disequilibrium (LD) and haplotype analysis, functional annotation, and literature reports. The Epigen Consortium was assessed to observe the frequency of the variants and haplotypes among the Brazilian samples. From the 3 candidate SNPs, only the rs11264359 did not match the ANNOVAR criteria (dashed line). B) Principal Component Analysis (PCA) of the PKLR SNPs in the populations of the 1000 Genomes Project. We used variants from a region of 10,000 bp upstream and downstream of the gene loci (chr1:155,259,084–155,271,225 –GRCh37/hg19) to observe the clusters displayed by each Principal Component (PC). Then, we evaluated the SNPs with the 30 highest scores (“SNP weight”) for the PC1 and, comparatively with the other analysis, we selected three candidate SNPs, given in S1 Table. EUR: Europeans; AFR: Africans and AMR: Native-Americans. (TIFF) [file pntd.0009434.s002.tiff]

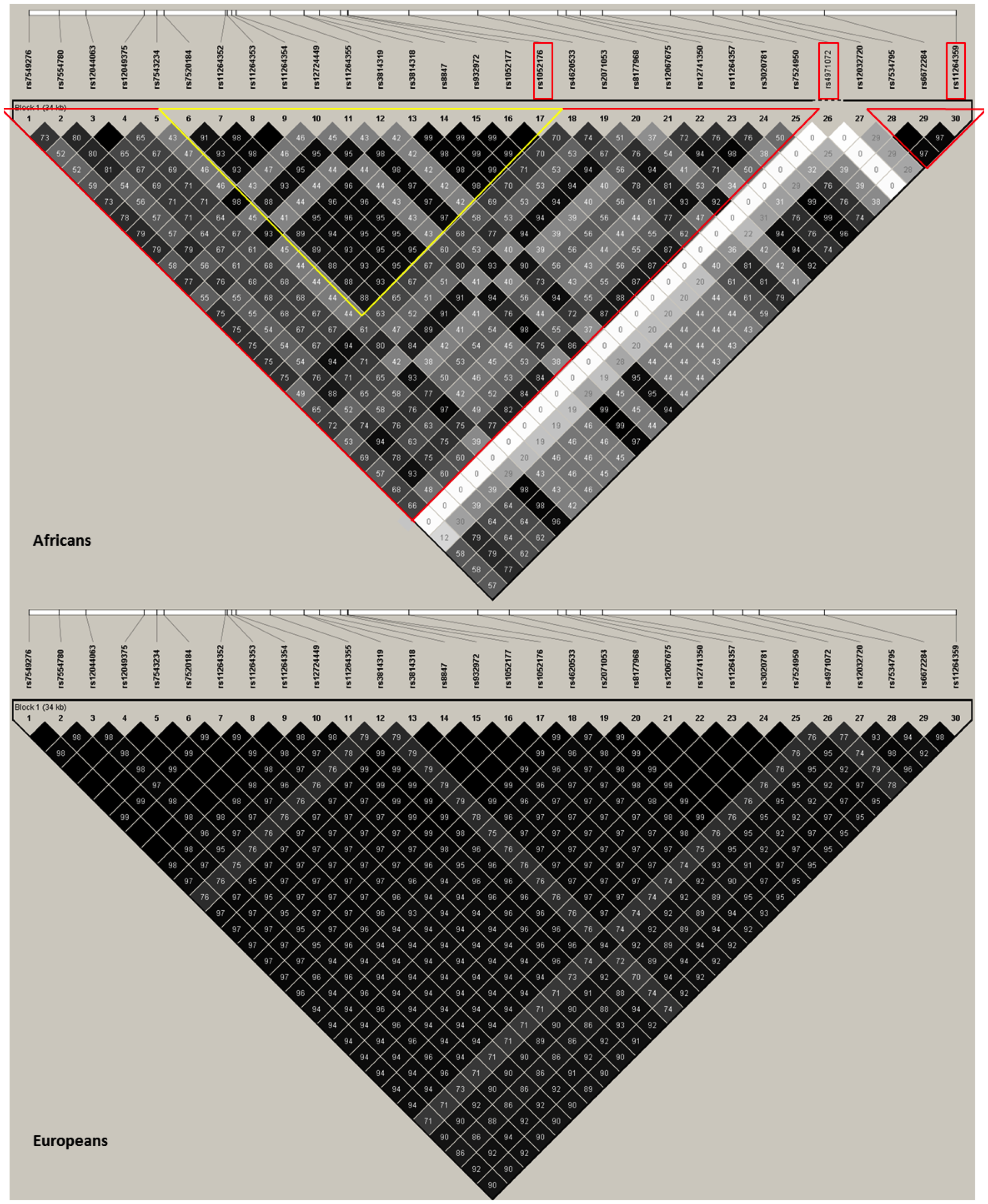

Supplement: S2 Fig — LD plot (r2*100) of the 30 top variants of the PKLR gene covering representative blocks of the region in the 1000 Genomes populations. In red, we observe the blocks represented by the tag SNPs detached in Africans and, in yellow, we highlighted the conserved block in high LD under selection by the xpEHH. (TIFF) [file pntd.0009434.s003.tiff]

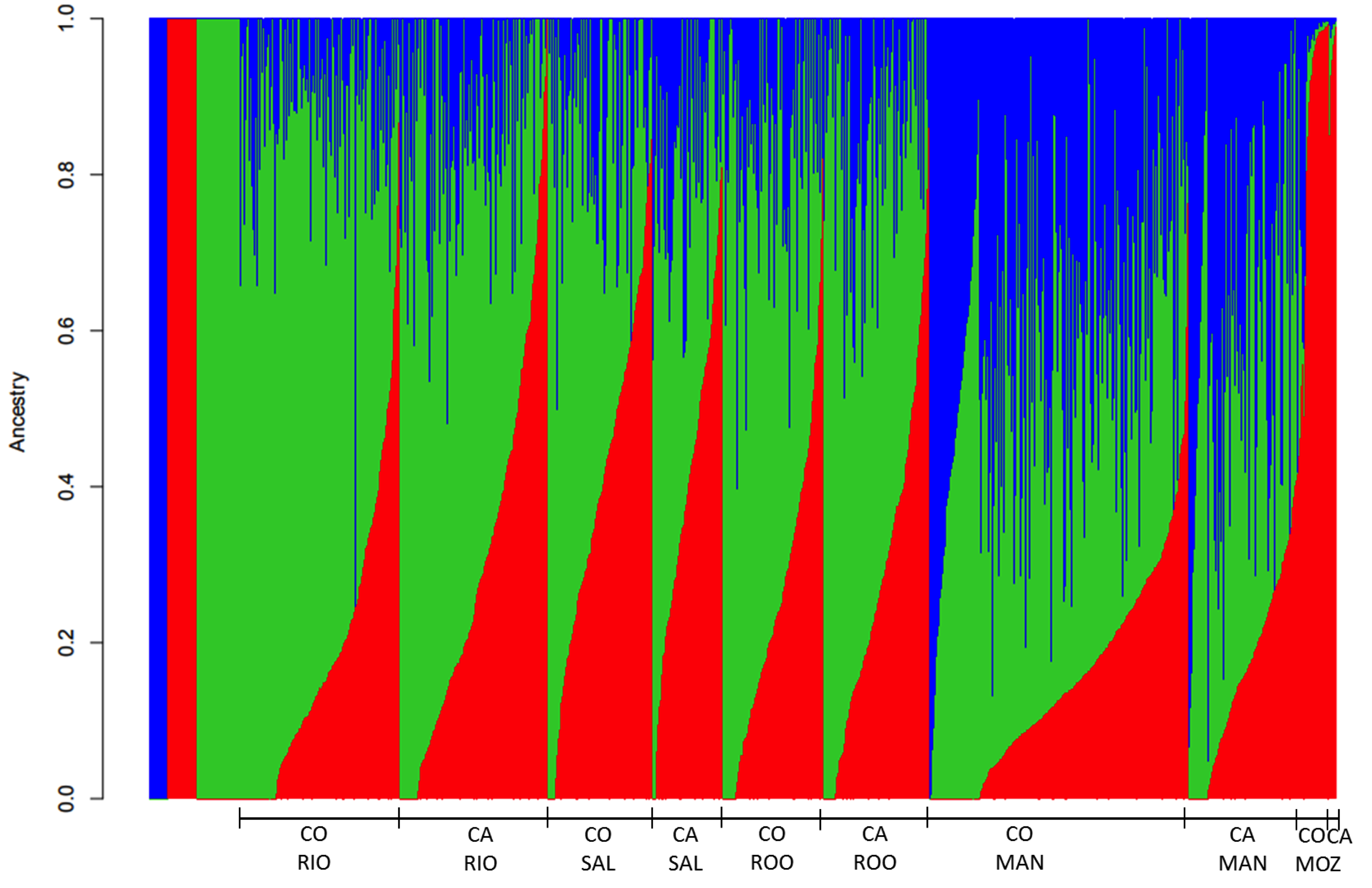

Supplement: S3 Fig — Parental populations from the HGDP-CEPH are represented in the blue (Native-American ancestry), red (African ancestry) and green bars (European ancestry). CO = Controls; CA = Cases; RIO = Rio de Janeiro; SAL = Salvador; ROO = Rondonópolis; MAN = Manaus and MOZ = Mozambique. (TIFF) [file pntd.0009434.s004.tiff]

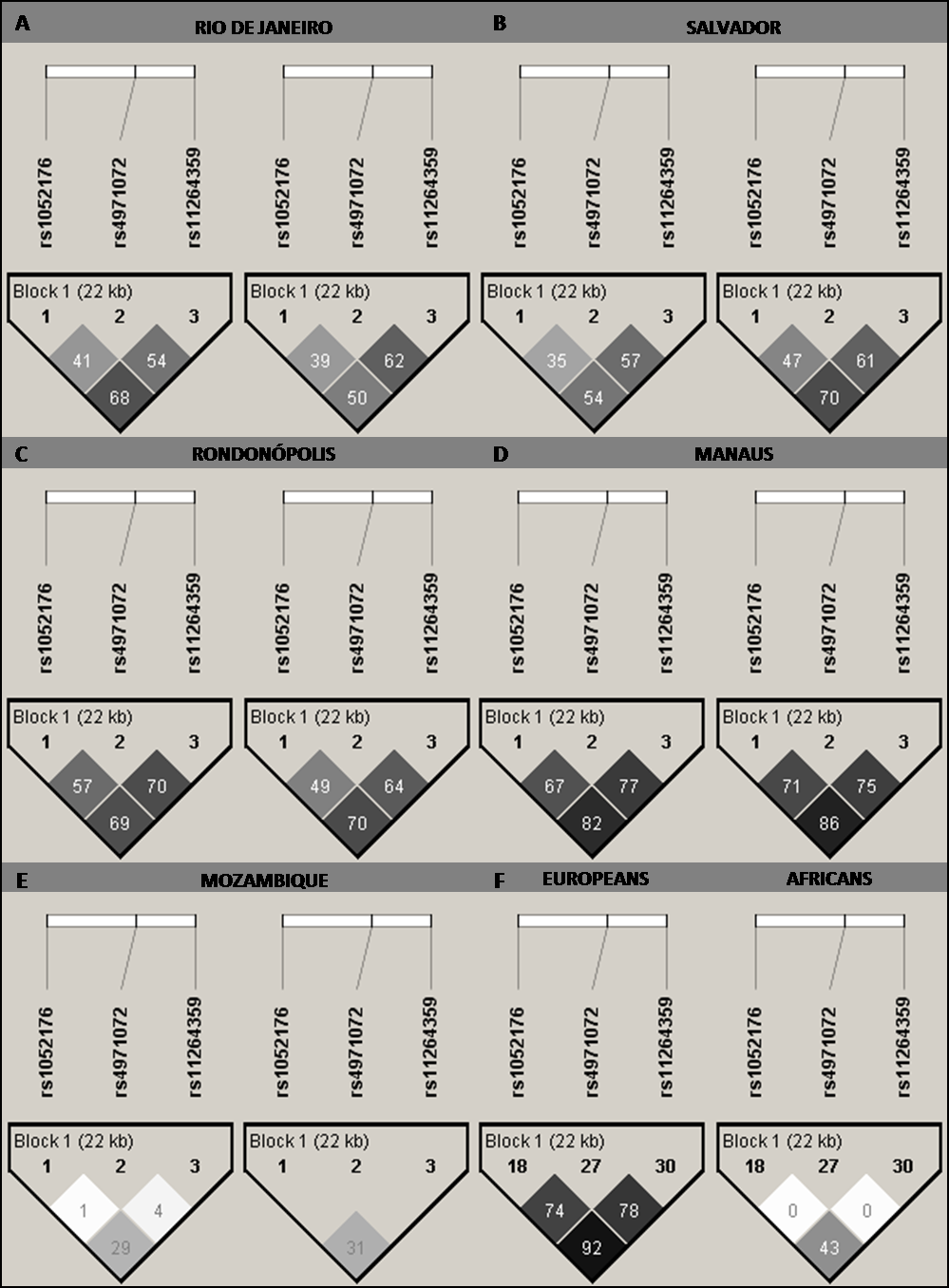

Supplement: S4 Fig — LD were calculated in r2. A-E) First and second LD plots in each population represents LD in controls and patients, respectively. F) LD in healthy European and African individuals from the 1000 Genomes Project. (TIF) [file pntd.0009434.s005.tif]

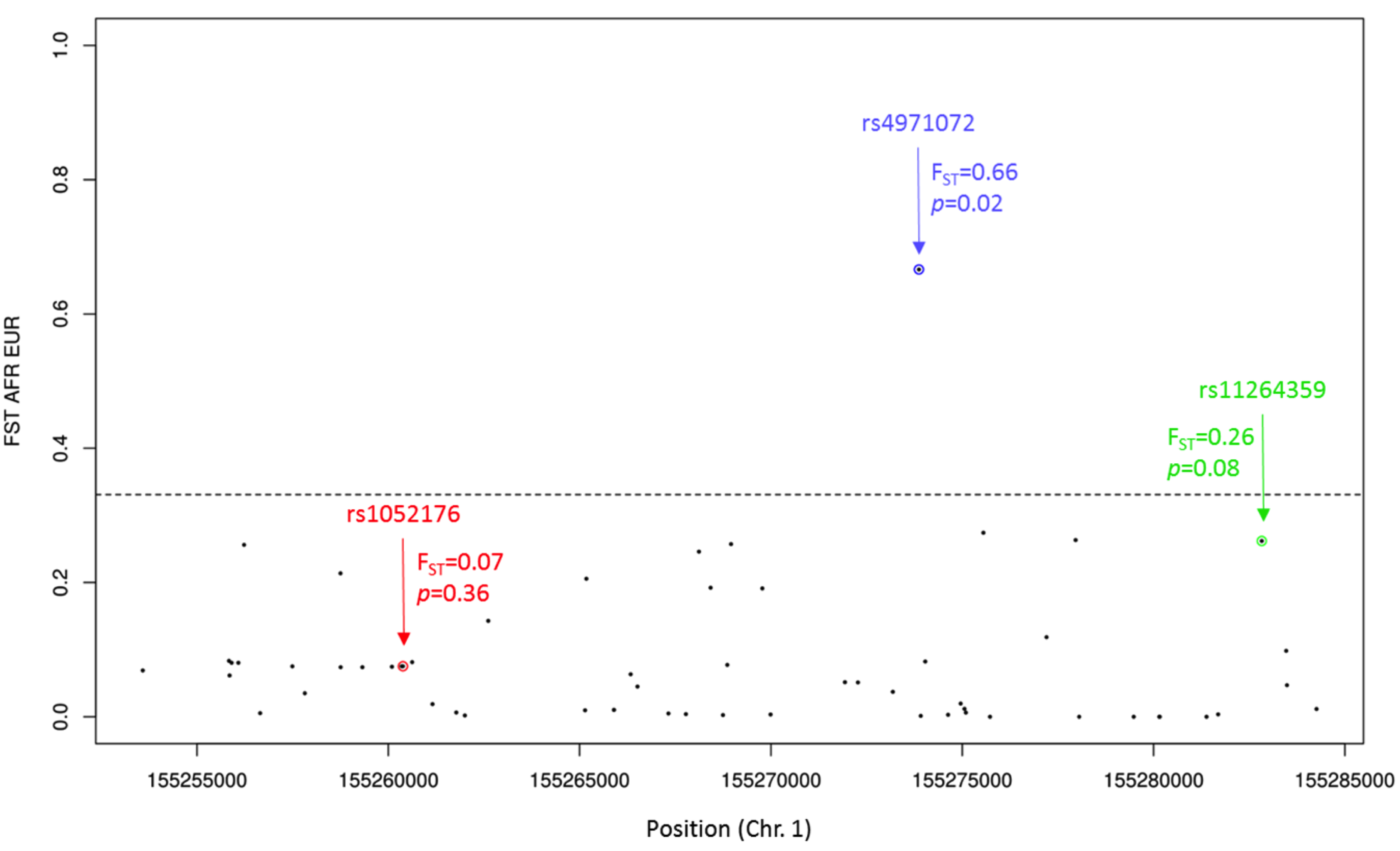

Supplement: S5 Fig — FST of 60 SNPs range in the PKLR genomic region between Africans (AFR) and Europeans (EUR) highlighting the FST and the empirical p values of the SNPs associated with mycobacteria. The dashed line represents the 95% quantile of the FST distribution along the chromosome 1. Red: rs1052176; blue: rs4971072; green: rs11264359. (TIFF) [file pntd.0009434.s006.tiff]

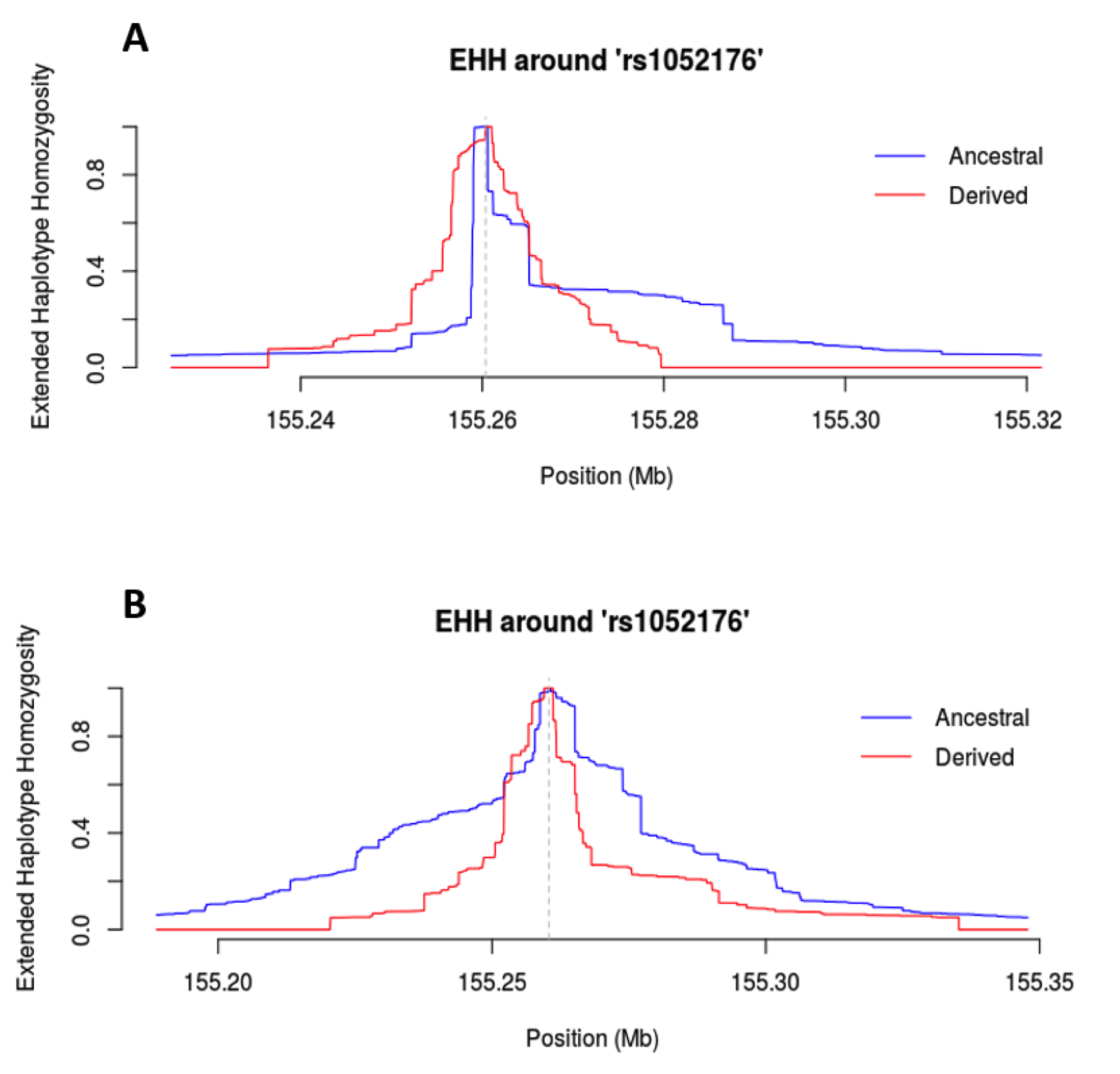

Supplement: S6 Fig — Decay of haplotypes (EHH) from the SNP core in Africans (A) and Europeans (B) from the 1000 Genomes Project. Horizontal lines are haplotypes, SNP positions are marked by the x-axis and the core SNP (rs1052176) position is represented by the dotted line. Blue indicates the EHH decay of the ancestral allele and red indicates the EHH decay of the derived allele. When the core SNP is neutral, the haplotype homozygosity decays at similar rates for both ancestral and derived alleles. When the derived alleles are favored, the haplotype homozygosity decays much slower for the derived alleles than for the ancestral alleles. (TIFF) [file pntd.0009434.s007.tiff]

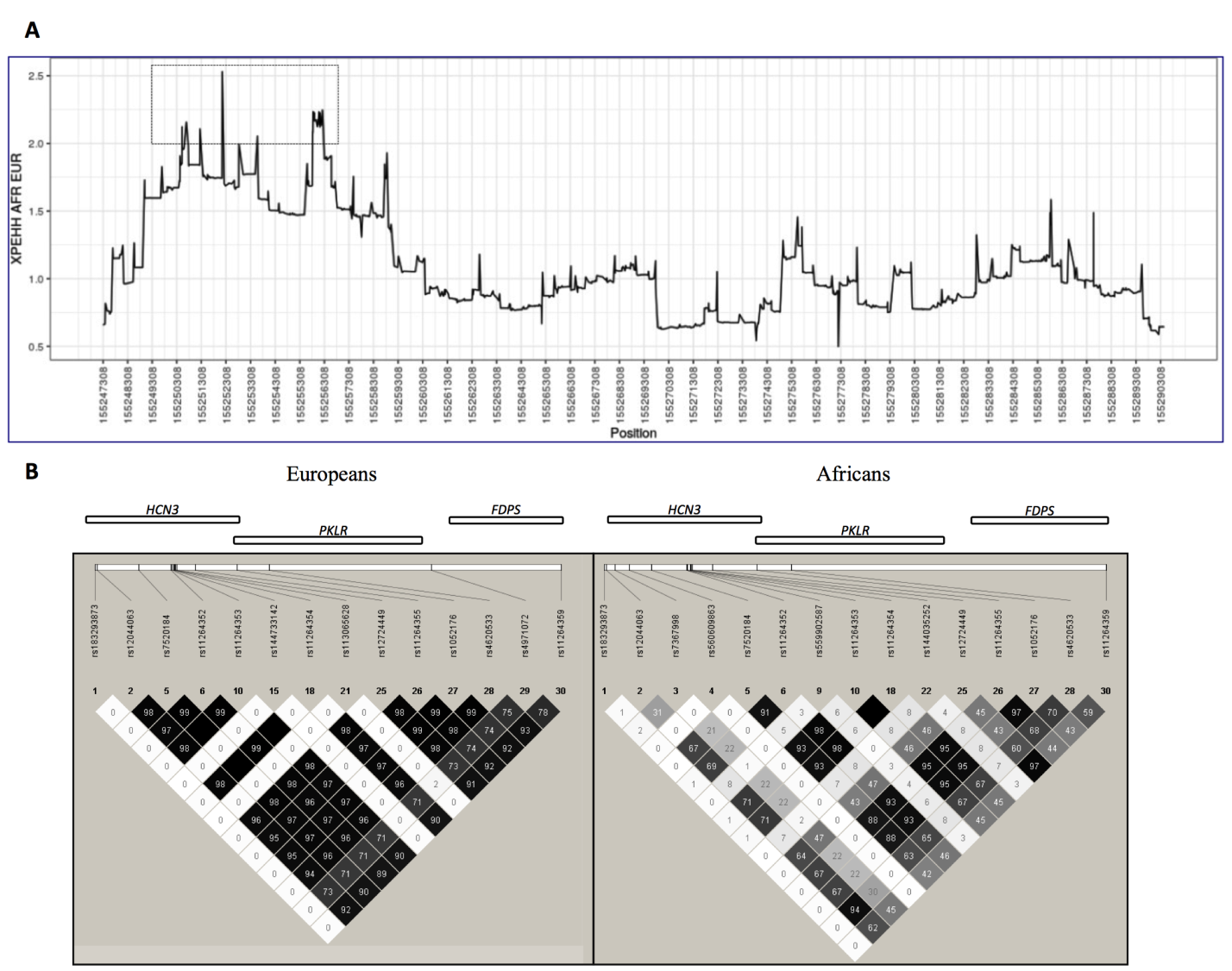

Supplement: S7 Fig — A) The signal (>|2.00|) of a sweep was observed for 25 SNPs in the HCN3 gene across 1060 variants covering HCN3, PKLR and FDPS genes in Europeans. B) Linkage disequilibrium (r2*100) between the SNPs within the HCN3, PKLR and FDPS genes in Europeans and Africans from the 1000 Genomes Project. (TIF) [file pntd.0009434.s008.tif]

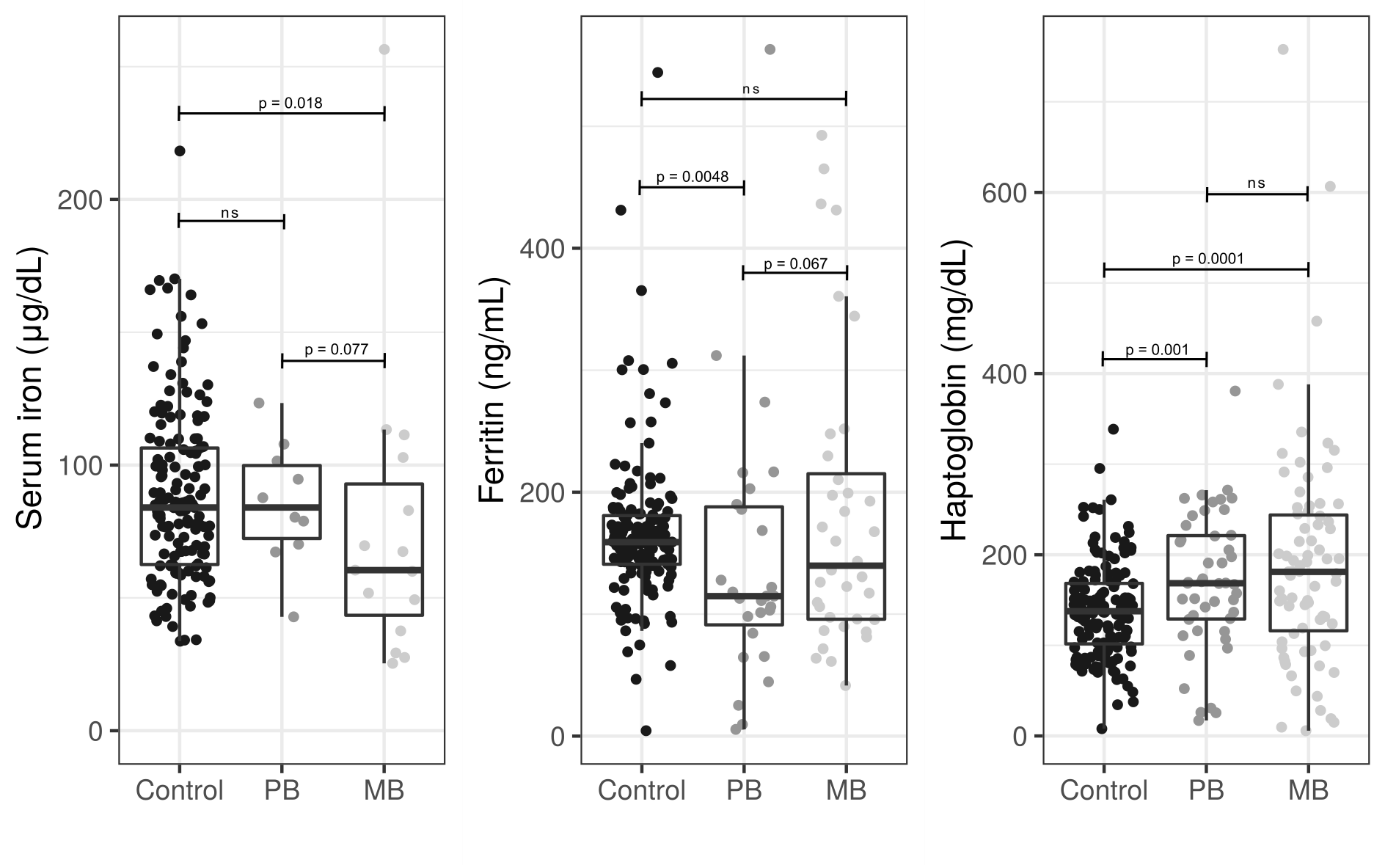

Supplement: S8 Fig — Bars represent the median of serum protein levels in each group adjusted by gender. Analysis were performed using Kruskal-Wallis test (p<0.05). Control = Healthy individuals; PB = Paucibacillary leprosy patients; MB = Multibacillary leprosy patients and ns = non-significant. (TIF) [file pntd.0009434.s009.tif]

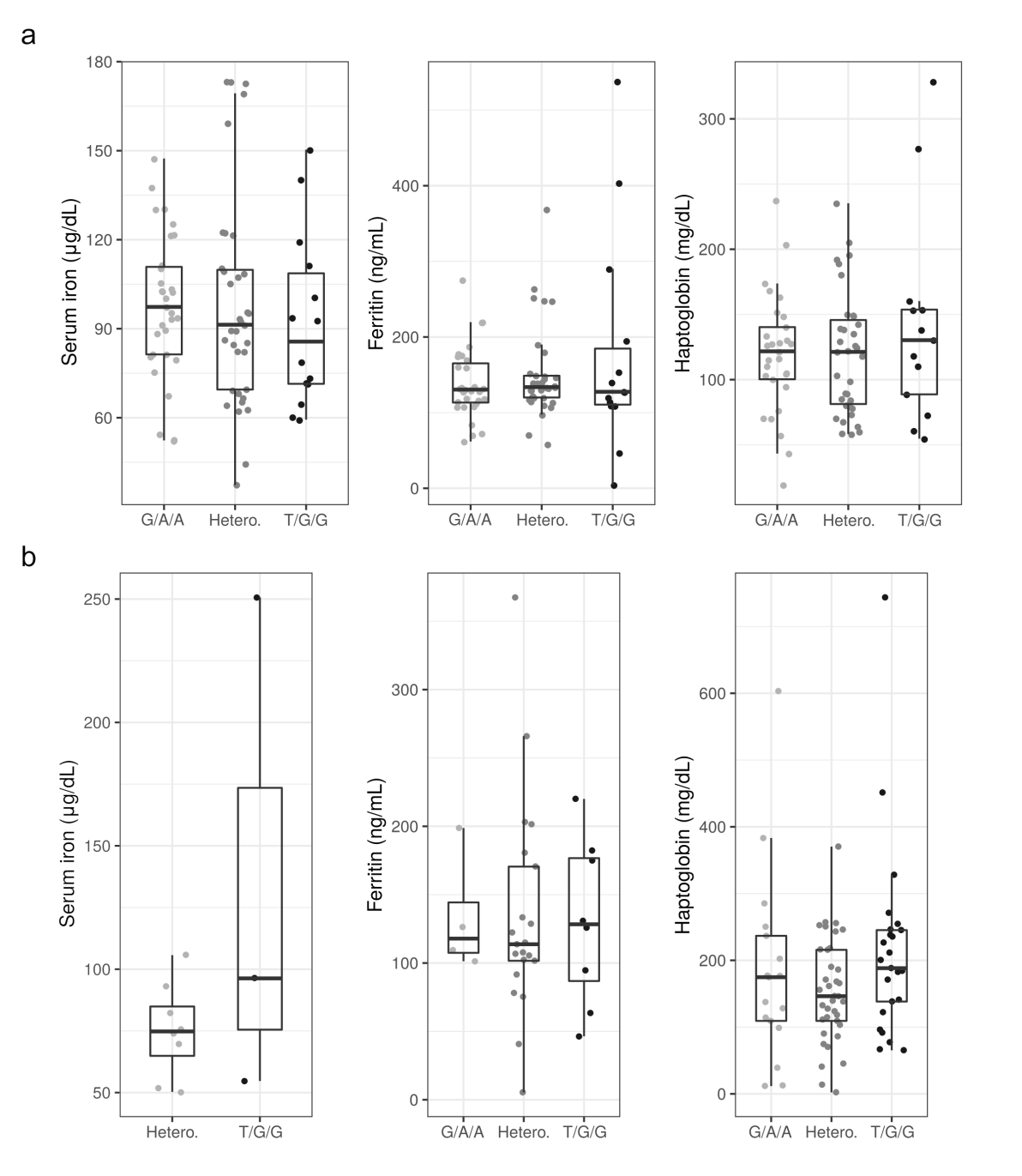

Supplement: S9 Fig — Bars represent the median adjusted by gender. G/A/A = Protection haplotype; Hetero. = Haplotype of the heterozygous and T/G/G = Risk haplotype from heterozygous individuals for the SNPs rs1052176, rs4971072 and rs11264359. Analysis were performed using Kruskal-Wallis test (p<0.05). A) Measurements in healthy subjects and B) Measurements among leprosy cases. (TIF) [file pntd.0009434.s010.tif]

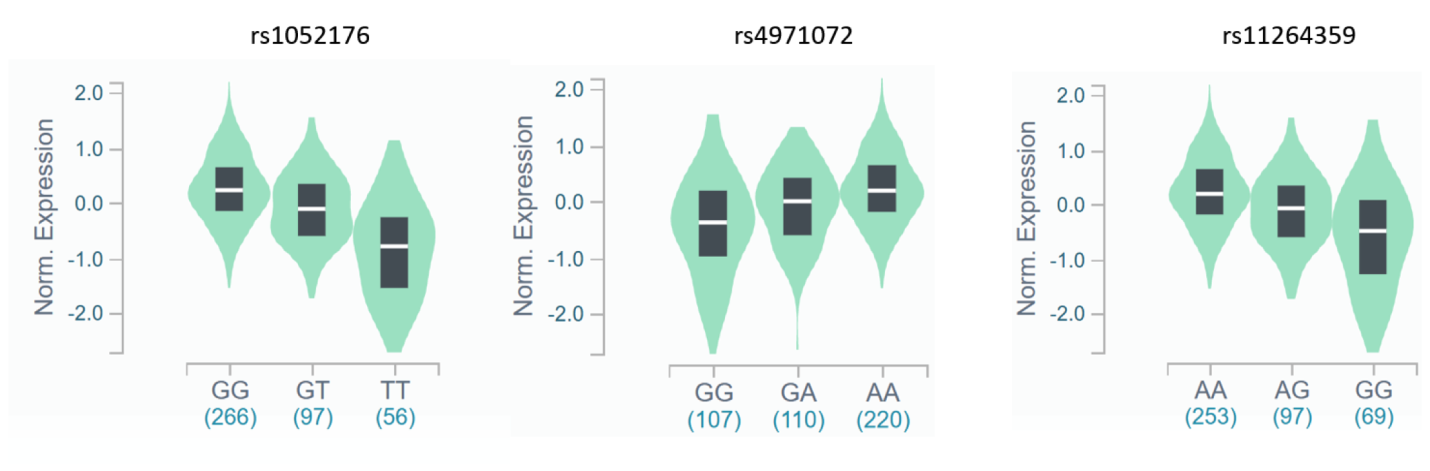

Supplement: S10 Fig — Median of the PKLR expression in the violin plot according to the genotype of each SNP. Number of individuals is represented in parentheses. Data were obtained of nerve biopsies according to the GTEx project, where the SNPs are eQTLs for the PKLR (rs1052176 p = 4.5x10^-29^, rs4971072 p = 2.9x10^-19^ and rs11264359 p = 1.1x10^-22^) and HCN3 (rs1052176 p = 1.4x10^-53^, rs4971072 p = 2.1x10^-40^ and rs11264359 p = 3.3x10^-48^) genes and with minor significance for the GBAP1 (rs1052176 p = 6.2x-10, rs4971072 p = 1.8x10^-10^ and rs11264359 p = 2.2x10^-10^), RIT (rs1052176 p = 4.0x10^-19^, rs4971072 p = 1.9x10^-12^ and rs11264359 p = 4.7x10^-11^) and FAM189B (rs1052176 p = 4.0x10^-5^, rs4971072 p = 1.1x10^-6^ and rs11264359 p = 1.8x10^-5^) genes. (TIF) [file pntd.0009434.s011.tif]
